# Celestial neuromorphics based on ferroelectric gallium oxide

**DOI:** 10.1093/nsr/nwag362

**Published:** 2026-06-12

**Authors:** Ke Xu, Zhannan Guan, Mengjiao Pei, Yurong Luo, Yonghao Zhu, Xinxin Yu, Lin Hao, Songhao Gu, Jiahao Yao, Zhanhua Li, Xinyi Pei, Yuhao Zhang, Han Wang, Changjin Wan, Qing Wan, Rong Zhang, Jiandong Ye

**Affiliations:** School of Electronic Science and Engineering, National Laboratory of Solid-State Microstructures, Nanjing University, Nanjing 210023, China; School of Electronic Science and Engineering, National Laboratory of Solid-State Microstructures, Nanjing University, Nanjing 210023, China; School of Electronic Science and Engineering, National Laboratory of Solid-State Microstructures, Nanjing University, Nanjing 210023, China; Department of Applied Physics, The Hong Kong Polytechnic University, Hong Kong 999077, China; School of Electronic Science and Engineering, National Laboratory of Solid-State Microstructures, Nanjing University, Nanjing 210023, China; State Key Laboratory of Superlattices and Microstructures, Institute of Semiconductors, Chinese Academy of Sciences, Beijing 100083, China; CETC Key Laboratory of Carbon-based Electronics, Nanjing Electronic Devices Institute, Nanjing 210016, China; School of Electronic Science and Engineering, National Laboratory of Solid-State Microstructures, Nanjing University, Nanjing 210023, China; School of Electronic Science and Engineering, National Laboratory of Solid-State Microstructures, Nanjing University, Nanjing 210023, China; School of Electronic Science and Engineering, National Laboratory of Solid-State Microstructures, Nanjing University, Nanjing 210023, China; School of Electronic Science and Engineering, National Laboratory of Solid-State Microstructures, Nanjing University, Nanjing 210023, China; School of Electronic Science and Engineering, National Laboratory of Solid-State Microstructures, Nanjing University, Nanjing 210023, China; Department of Electrical and Electronic Engineering, University of Hong Kong, Hong Kong 999077, China; Department of Electrical and Electronic Engineering, University of Hong Kong, Hong Kong 999077, China; School of Electronic Science and Engineering, National Laboratory of Solid-State Microstructures, Nanjing University, Nanjing 210023, China; Yongjiang Laboratory (Y-LAB), Ningbo 315202, China; Yongjiang Laboratory (Y-LAB), Ningbo 315202, China; School of Electronic Science and Engineering, National Laboratory of Solid-State Microstructures, Nanjing University, Nanjing 210023, China; School of Electronic Science and Engineering, National Laboratory of Solid-State Microstructures, Nanjing University, Nanjing 210023, China

**Keywords:** gallium oxide, ferroelectric device, in-sensor neuromorphic electronics

## Abstract

Efficient recognition of celestial activities demands hardware that can operate with high efficiency and robustness in radiation-rich space environments. Ultra-wide bandgap (UWBG) semiconductors are well-suited for such environments, but conventional UWBG transistors are not inherently compatible with advanced computing functions. To address this limitation, here, we report a *κ*-phase gallium oxide (*κ*-Ga_2_O_3_) based in-sensor reservoir computing system (*κ*-ISRC), which incorporates deep ultraviolet sensing, memory, and neuromorphic computation for celestial activity recognition. A ferroelectric high-electron-mobility transistor is fabricated by exploiting polarization switching of *κ*-Ga_2_O_3_ through atomic sliding mechanisms. The Al_2_O_3_/*κ*-Ga_2_O_3_ dielectric/ferroelectric gate stack provides negative-capacitance effect, supporting configurable memory operations. Furthermore, the device can maintain its performance over a wide temperature range (from −270 to 210°C) and under ion irradiation with an average flux of 1×10^4^ cm^−2^ s^−1^. Leveraging these device features, the celestial neuromorphic system achieves up to 95% classification accuracies across diverse astrophysical events, including solar flares, cosmic-ray bursts, and pulsar emissions. This work establishes UWBG ferroelectric semiconductors as a multifunctional platform for energy-efficient in-sensor neuromorphic electronics for aerospace and deep-space applications.

## Teaser

A celestial neuromorphic system is developed based on κ-phase gallium oxide, exhibiting high robustness to the space environment and high efficiency in analyzing dynamic astrophysical events.

## INTRODUCTION

Light curve analysis, which captures time-resolved brightness variations of celestial objects across multiple wavebands, is a cornerstone technique in observational astronomy for probing dynamic astrophysical phenomena [1,2]. Precise extraction and recognition of these temporal signals are pivotal for disaster monitoring and for classifying cosmic events such as stellar flares, eclipsing binaries, cataclysmic variables, and irregular variable stars [3]. Deep-ultraviolet (DUV) light detection plays a particularly important role, as many in capturing astrophysical activities manifest strongly in this spectral range and are primarily observed using space-based telescopes [4–6]. Conventional recognition of light curves relies on von Neumann computing architectures, where sensing, memory, and processing are separated [7,8]. Such separation results in increased latency and energy consumption in light curve recognition, and these limitations are especially critical in space applications with constrained power budgets and payload volumes [9]. Therefore, breaking the fundamental trade-off between efficiency, compactness, and radiation robustness of celestial signal processing hardware is of growing importance.

Physical reservoir computing (RC) has emerged as a promising low-power computing paradigm for temporal data processing [10–12]. It requires training only the output layer while leveraging the intrinsic dynamics of physical systems to perform complex transformations on input time series. In-sensor reservoir computing further integrates sensing and computing functions within a single hardware platform, significantly reducing energy consumption and response latency. For example, SnS-based in-sensor RC achieved 91% accuracy in real-time language classification with minimal training cost [13], and a GaO_*x*_/TaO_*x*_/HfO_*y*_ hybrid system enabled efficient latent fingerprint recognition [14]. In the latter, persistent photoconductivity (PPC) in amorphous GaO_*x*_ facilitated nonlinear transformation, while the memristor array enabled multilevel memory. However, reliance on disparate materials and device types increases integration complexity and raises compatibility concerns, limiting scalability.

Aerospace applications place stringent demands on radiation-tolerant electronic materials. Ultra-wide bandgap (UWBG) semiconductors, such as gallium oxide (Ga_2_O_3_), are well-suited for such environments because of their large bandgap (∼4.9 eV for Ga_2_O_3_) [15], high breakdown electric field (∼8 MV/cm for Ga_2_O_3_), and a high threshold displacement energy (∼28 eV for Ga_2_O_3_) against radiation [16]. However, conventional UWBG transistors are not inherently suitable for computing applications due to challenges such as high Ohmic contact resistance and low carrier mobility [17,18]. To address this limitation, integrating ferroelectric functionality into UWBG semiconductors provides a promising pathway, enabling new device architectures that combine radiation robustness with efficient neuromorphic computation.

Among its polymorphs (α, β, γ, δ, κ), *κ*-Ga_2_O_3_ is predicted to possess ferroelectricity and has demonstrated potential for multilevel memory operation [19,20]. These properties make *κ*-Ga_2_O_3_ uniquely suited for unifying DUV sensing, nonlinear transformation, and memory within a single platform, all of which are key elements for reconfigurable in-sensor RC. Yet, direct experimental evidence of ferroelectric switching in *κ*-Ga_2_O_3_ remain scarce [19,21]. Although *κ*-Ga_2_O_3_/GaN ferroelectric FETs have been proposed, polarization reversal in *κ*-Ga_2_O_3_ itself has not been conclusively demonstrated for device applications [22]. This gap hinders the full exploitation of its capabilities for neuromorphic computing, particularly in radiation-intense environments like outer space.

Here, we demonstrate a *κ*-Ga_2_O_3_-based in-sensor reservoir computing (*κ*-ISRC) system for efficient recognition of celestial activities. The system integrates a *κ*-Ga_2_O_3_-based DUV sensor with *κ*-Ga_2_O_3_-gated AlGaN/GaN ferroelectric high-electron-mobility transistors (FeHEMT), enabling in-sensor reservoir computing. We report direct observation of ferroelectric polarization switching in *κ*-Ga_2_O_3_ via atomic sliding along [100]. The resultant FeHEMT supports both short-term and long-term memory (LTM) modes by programing channel conductivity through carrier trapping/detrapping processes and ferroelectric polarization switching, respectively. The device also shows great potential for celestial applications, as it can maintain its performance over a wide temperature range (from −270 to 210°C) and under ion irradiation with an average flux of 1×10^4^ cm^−2^s^−1^. The *κ*-ISRC system employs the *κ*-Ga_2_O_3_ sensor for DUV detection and collects reservoir states through the FeHEMT operating in short-term mode (S-mode). The resulting reservoir states are then fed into the FeHEMT in long-term mode (L-mode), functioning as the output layer for label inference. This *κ*-ISRC system achieves up to 95% accuracy in recognizing four types of celestial activities, demonstrating its potential for energy-efficient, radiation-resilient neuromorphic hardware in astrophysical and aerospace applications.

## RESULTS AND DISCUSSION

### *κ*-ISRC system

**Figure 1.**
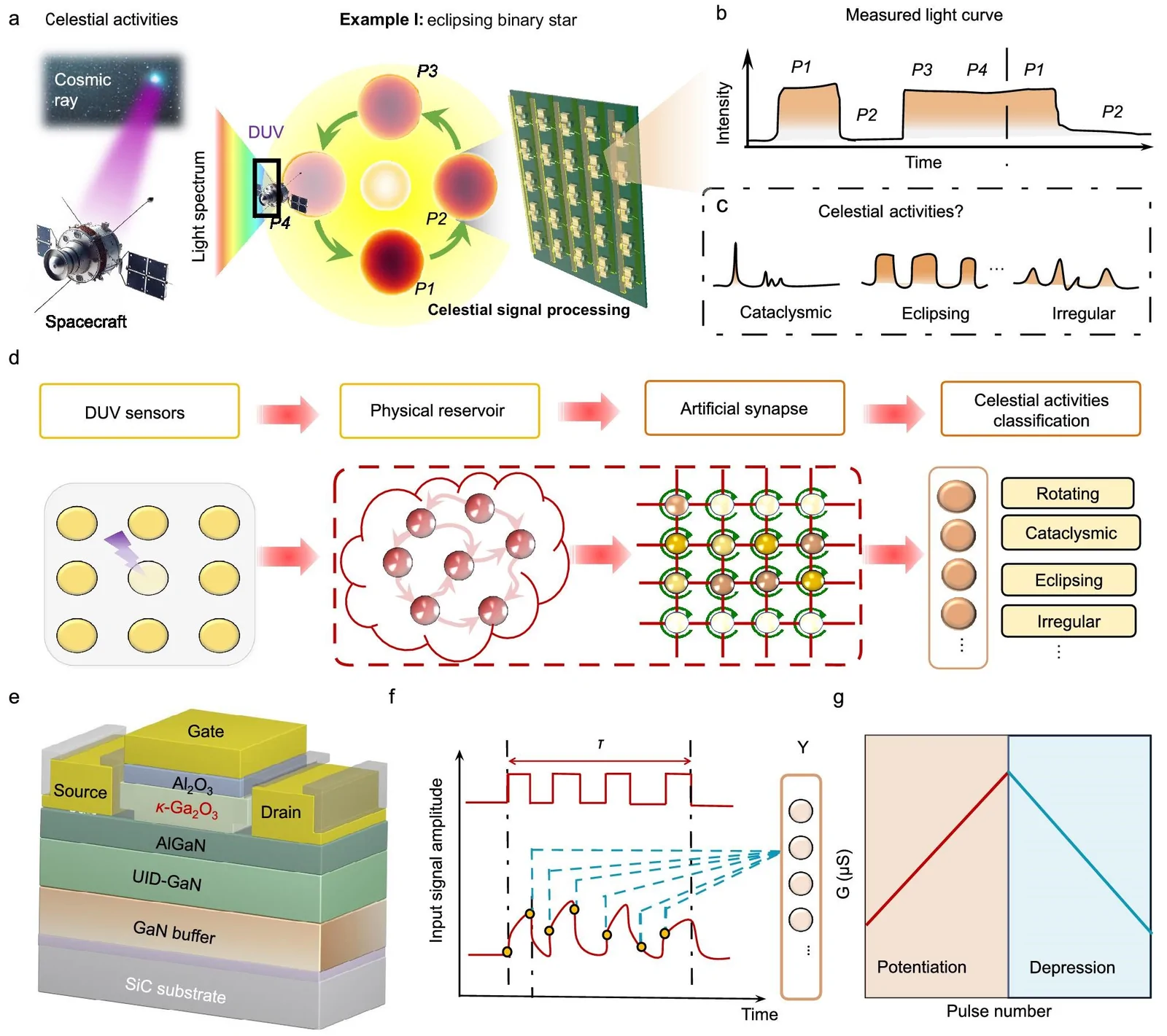
Schematic overview of the *κ*-Ga_2_O_3_-based reconfigurable in-sensor reservoir computing (*κ*-ISRC) system. (a) Detection and signal processing of eclipsing binary star system based on light curves from cosmic ray. (b) Schematic of a light curve from an eclipsing binary system. (c) Representative light curves of different celestial activities. (d) Workflow of the *κ*-ISRC system from light detection using a *κ*-Ga_2_O_3_ DUV sensor to reservoir state collection and label inference through the FeHEMT reservoir and the output layer of a fully connected neural network. (e) Schematic structure of the *κ*-Ga_2_O_3_-gated FeHEMT. (f) Temporal dynamics of reservoir computing by applying square pulses. (g) Schematic of synaptic weight updating in the *κ*-Ga_2_O_3_-gated FeHEMT.

The conceptual framework for celestial activity recognition aboard a spacecraft is illustrated in Fig. 1a. Temporal brightness variations of celestial objects, known as light curves, are fundamental footprints of dynamic astrophysical events. Take an eclipsing binary system as an example (example I, Fig. 1a), periodic dimming occurs as the companion star transits across the primary star, modulating the observed DUV intensity. Therefore, DUV variations contained in the light curve, reflect astrophysical information (Fig. 1b), such as eclipse event (P2) and non-eclipse intervals (P1, P3, and P4). In this case, distinct light curves can serve as ‘identifiers’ of celestial activities (such as cataclysmic variables, eclipsing binaries, and irregular variable stars, as shown in Fig. 1c).

To process such signals, we developed a reconfigurable in-sensor RC system comprising three functional blocks: a DUV sensor for celestial radiation detection, a reservoir for high-dimensional encoding of time-series data, and an output layer based on artificial synapses for label inference (Fig. 1d). In this system, the DUV component contained in light curves can be detected and converted into electrical conductance/current signals by the *κ*-Ga_2_O_3_-based DUV sensor. *κ*-Ga_2_O_3_ exhibits strong responsivity below 260 nm, making it ideal for DUV sensing. These sensing signals are transformed into nonlinear temporal patterns and applied on the *κ*-Ga_2_O_3_-based FeHEMTs which function as physical reservoir to collect reservoir states. These reservoir states are then passed through *κ*-Ga_2_O_3_-gated FeHEMTs which function as artificial synapses for final classification [23–25]. The structure (Fig. 1e) of such bifunctional FeHEMT consists of an AlGaN/GaN heterostructure supporting a two-dimensional electron gas (2DEG) channel, and a *κ*-Ga_2_O_3_/Al_2_O_3_ gate dielectric stack for ferroelectric control. Fabrication processes are detailed in Method and Fig. S1. Specifically, the critical geometrical parameters of the device were defined by a source-drain spacing of 16 μm and a gate electrode length of 10 μm.

The device exhibits reconfigurable memory behavior: under sub-coercive fields of ferroelectric *κ*-Ga_2_O_3_ gate, charge trapping/de-trapping at interfacial states dominates, enabling short-term memory (STM) function suitable for temporal encoding in the reservoir [26,27]. Such STM property can meet requirements of physical reservoir for mapping time-series data into high-dimensional space in a nonlinear manner, as shown in Fig. 1f. Under fields exceeding the coercive threshold, ferroelectric polarization switching governs conduction, producing L-mode with stable multilevel conductance states that emulate synaptic potentiation and depression, as shown in Fig. 1g.

### Ferroelectric polarization switching of κ-Ga_2_O_3_

**Figure 2.**
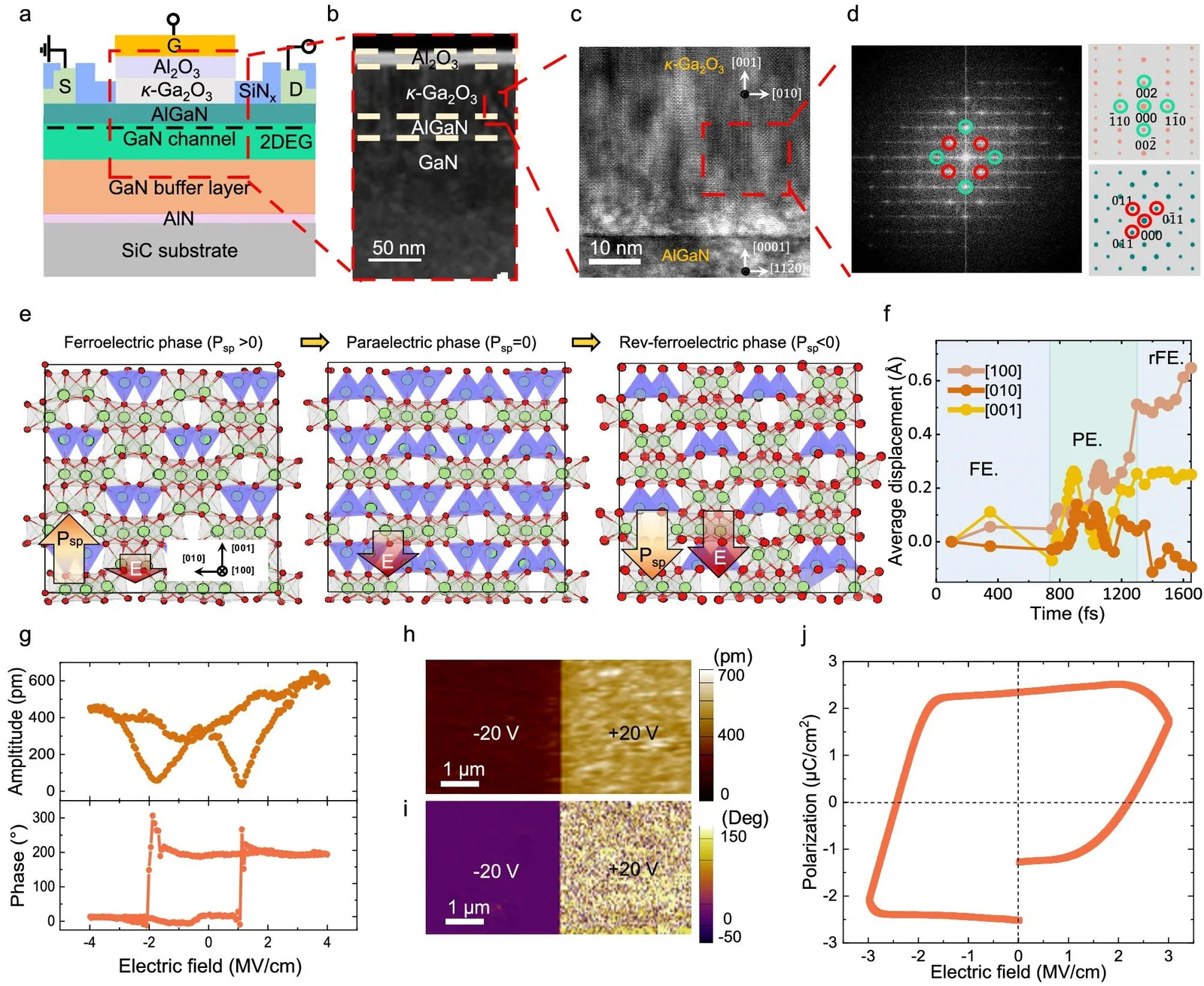
Structural characterization and ferroelectric polarization properties of *κ*-Ga_2_O_3_ epilayer on an AlGaN/GaN heterostructure. (a) Cross-sectional schematic of *κ*-Ga_2_O_3_-gated FeHEMT. (b) Cross-sectional STEM image of gate and channel structures. (c) HRTEM image of *κ*-Ga_2_O_3_/AlGaN interface. (d) Electron diffraction and simulated patterns of *κ*-Ga_2_O_3_, indicating rotational domains along [100] and [110]. (e) Ferroelectric flipping of *κ*-Ga_2_O_3_ simulated by AIMD. (f) Average atomic displacements along three crystallographic axes during AIMD simulation. (g) PFM hysteresis loops of amplitude and phase, verifying ferroelectricity of *κ*-Ga_2_O_3_. (h) PFM amplitude and (i) phase mappings of out-of-plane polarizations in *κ*-Ga_2_O_3_ under a sequential bidirectional DC bias of ±20 V. (j) Polarization–electric-field loops of the *κ*-Ga_2_O_3_ capacitor, with *P*_r_, *P*_S_, and *E*_c_, denoting remnant polarization, saturation polarization and coercive field, respectively.

The FeHEMT gate stack integrates a *κ*-Ga_2_O_3_/Al_2_O_3_ ferroelectric/dielectric (FE/DE) bilayer on AlGaN barrier layer, with the channel formed by the polarization-induced 2DEG at the AlGaN/GaN interface [28], as shown in Fig. 2a. The Al_2_O_3_ DE layer mitigates depolarization of *κ*-Ga_2_O_3_ and stabilizes negative capacitance [29,30], while a thin SiN_*x*_ passivation layer reduces gate-to-source/drain leakage currents. Cross-sectional scanning transmission electron microscopy (STEM) analyses revealed uniform *κ*-Ga_2_O_3_ (50 nm), Al_2_O_3_ (10 nm), and AlGaN (25 nm) layers with sharp interfaces (Fig. 2b), corroborated by energy-dispersive X-ray spectrometry (EDS) mapping (Fig. S2b). X-ray diffraction analysis evidences single-crystalline *κ*-Ga_2_O_3_ (001) without secondary phases (Fig. S3a). A narrow rocking curve width of 0.2° (Fig. S3b) and root-mean-square surface roughness of 1.2 nm (Fig. S3c) further indicate high-quality selective-area epitaxy of *κ*-Ga_2_O_3_ on AlGaN (0001) [31]. High-resolution transmission electron microscope (HRTEM) along *κ*-Ga_2_O_3_ [100] and AlGaN [1${\bar{1}}$ 00] (Fig. 2c) confirms coherent crystalline domains and sharp interfaces. The corresponding fast Fourier transform diffraction pattern (Fig. 2d) indicates the presence of three in-plane rotational domains separated by 120°, each adopting distinct orientations aligned with the [100] [110] and [${\bar{1}}$10] zone axes, which are consistent with orthorhombic symmetry and previous simulations [32]. Ab initio molecular dynamics (AIMD) simulations showed that polarization reversal arises from Ga migration between octahedral and tetrahedral sites parallel to the c-plane under an external electric field along the $[ {00\bar{1}} ]$ direction. Lattice snapshots extracted from a 1700 fs AIMD trajectory (Fig. 2e) shows transitions between the ferroelectric (FE), paraelectric (PE), and reversed-ferroelectric (rFE) states. The Ga atoms exhibit dominant displacement along [100] (0.65 Å), compared with only 0.25 and 0.09 Å along $[ {001} ]$ and $[ {010} ]$, respectively (Fig. 2f). This indicates that polarization switching is primarily mediated by motion perpendicular to the applied field. This polarization switching behavior bears resemblance to the mechanism reported in orthorhombic GaFeO_3_ (space group Pna2_1_), where polarization arises from anion displacement nearly perpendicular to the polar axis [33,34]. The calculated energy-polarization curve exhibits a double-well potential with a negative slope region, consistent with negative capacitance behavior, as shown in Fig. S4.

Ferroelectric switching was experimentally verified via piezoresponse force microscopy (PFM) in off-field mode [35]. The butterfly-shaped amplitude and hysteresis phase responses verify polarization reversal, with positive and negative coercive fields (*E*_c_) of approximately +1 and −2 MV/cm, respectively (Fig. 2g). PFM amplitude and phase mappings under sequential ±20 V poling showed clear contrast, evidencing switchable out-of-plane polarization (Fig. 2h and i). The polarization-electric field (P-E) loop of a *κ*-Ga_2_O_3_ capacitor reveals a remanent polarization (*P*_r_) of ∼2.5 μC/cm^2^ (Fig. 2j). Both *P*ᵣ and *E*_c_ increase nearly linearly with the applied electric field, further supporting field-driven ferroelectric switching, as shown in Fig. S5. Off-field hysteresis measurements (Fig. S6a and b) of local PFM hysteresis loops under various driving frequencies (1–10 Hz) and polarization-electric field loop (Fig. S6c) under various scanning frequencies (600–2500 Hz), indicate the possible dynamics should not be non-ferroelectric mechanisms such as electrostatic or capacitive processes.

### Characterization of ferroelectric high-electron-mobility transistor

**Figure 3.**
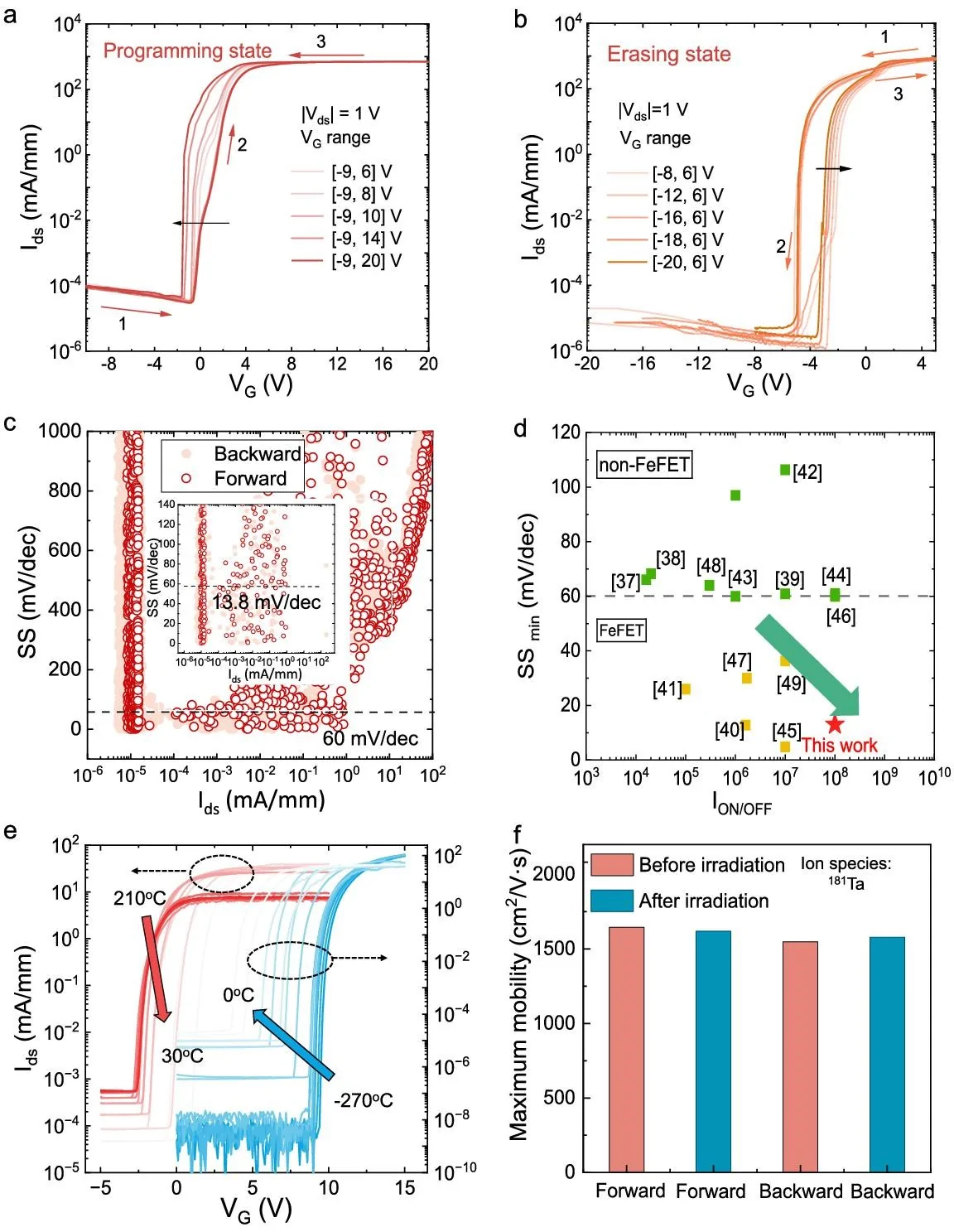
Electrical characterizations of *κ*-Ga_2_O_3_-gated FeHEMT. (a) Hysteresis curves of a *κ*-Ga_2_O_3_ gated FeHEMT under increasing positive gate voltage sweeps from −9.0 to +20 V (program states). (b) Hysteresis curves under increasing negative gate voltage sweeps from +6.0 to −20 V (erase states). (c) SS measured during forward and backward gate sweeps with a rate of 0.003 V/s, demonstrating SS below the Boltzmann limit of 60 mV/dec. (d) Benchmark plot of SS_min_ versus ION/OFF ratio of *κ*-Ga_2_O_3_-gated FeHEMT, compared with previous reported devices [39–51]. (e) Temperature-dependent transfer characteristics of the device. (f) Maximum mobilities of the κ-Ga_2_O_3_-gated FeHEMT before and after ^181^Ta heavy-ion irradiation.

The ferroelectric modulation of *κ*-Ga_2_O_3_-gated AlGaN/GaN FeHEMT were experimentally examined. Figure 3a shows the transfer characteristics at a source-drain bias (*V*_DS_) of 1.0 V under forward gate voltage sweeps (*V*_GS_ from −9 V to successively increasing positive values up to 20 V) followed by reverse sweeps. During forward sweeps, the threshold voltage (*V*_TH_) remains nearly constant, whereas reverse sweeps present a progressive negative shift in *V*_TH_ as the maximum positive *V*_GS_ increasing from 6 to 20 V, resulting in an increasing Δ*V*_TH_ up to 1.41 V at a drain current density (*I*_DS_) of 10^−2^ mA/mm. This behavior reflects gradual polarization programing in *κ*-Ga_2_O_3_. To initialize full polarization, a +20 V bias was applied prior to the reverse sweeps. Subsequent cycling with increasing negative gate sweeps down to −20 V (Fig. 3b) results in negligible Δ*V*_TH_ during sweeps from positive to negative *V*_GS_; however, it induces a significant positive V_TH_ shift upon backward sweeps from negative to positive *V*_DS_, with Δ*V*_TH_ increasing from 0.2 to 1.03 V. This confirms effective ferroelectric depolarization and establishment of erasing states.

Notably, both Fig. 3a and b shows distinct counterclockwise hysteresis regardless sweeping directions, with high ON/OFF current ratios (*I*_ON/OFF_) of ∼10^8^. The corresponding output characteristics further highlight polarization-dependent current modulation, as shown in Fig. S7a and b. Devices in the poling-down state exhibit enhanced transconductance (395 mS/mm) and mobility (1550 cm^2^/V.s), compared to 293 mS/mm and 1250 cm^2^/V.s in the poling-up state, as shown in Fig. S8a–c.

The subthreshold swing (SS) that quantitatively describes the gate control over channel potential in the subthreshold regime, is defined by


(1) \begin{eqnarray*}
{\mathrm{SS}} = \frac{{\partial {{\mathrm{V}}}_{\mathrm{G}}}}{{\partial {{\log }}_{10}{I}_{{\mathrm{DS}}}}} = \frac{{\partial {{\mathrm{V}}}_{\mathrm{G}}}}{{\partial {{\mathrm{\psi }}}_{\mathrm{S}}}}\frac{{\partial {{\mathrm{\psi }}}_{\mathrm{S}}}}{{\partial {{\log }}_{10}{I}_{{\mathrm{DS}}}}}\ ,
\end{eqnarray*}

where ${{\mathrm{\psi }}}_{\mathrm{S}}$ is the channel potential; $\frac{{\partial {{\mathrm{\psi }}}_{\mathrm{S}}}}{{\partial {{\log }}_{10}{I}_{{\mathrm{DS}}}}}$ is the transconductance factor, and the body factor term, $\frac{{\partial {V}_{\mathrm{G}}}}{{\partial {{\mathrm{\psi }}}_{\mathrm{S}}}} = ( {1 + \frac{{{C}_{\mathrm{s}}}}{{{C}_{{\mathrm{ins}}}}}} )$, depends on the capacitances of channel (${C}_{\mathrm{s}})$ and gate (${C}_{{\mathrm{ins}}}$). At room temperature, the theoretical limit is 60 mV/dec (i.e. ${\mathrm{SS}}\geqslant\frac{{{\mathrm{ln}} 10 \cdot {{\mathrm{k}}}_{\mathrm{B}}{\mathrm{T}}}}{q}$). However, if ${C}_{{\mathrm{ins}}}$ becomes negative induced by ferroelectric gate layer, SS can fall below the limit. Figure 3c shows SS extracted from bidirectional transfer curves at a *V*_G_ sweep rate of 0.003 V/s, yielding an average SS (SS_ave_) of 50 mV/dec, and a minimum SS (SS_min_) of 13.8 mV/dec at *V*_G_ ranging −1.6 to −1.4 V, indicating negative capacitance effect of the *κ*-Ga_2_O_3_/Al_2_O_3_ gate dielectric stack.

The demonstrated sub-60 mV/dec switching surpasses conventional AlGaN/GaN HEMT without ferroelectric gating [36,37]. Similar architecture have recently been reported to exploit gate negative capacitance, breaking the fundamental trade-off between ON and OFF currents and surpassing the performance limits of Schottky GaN transistors with conventional dielectrics [38]. At higher sweep rates (e.g. 0.2 V/s), SS_min_ increases (Fig. S9), consistent with incomplete polarization switching. This is corroborated by *κ*-Ga_2_O_3_-based metal–oxide–semiconductor capacitors, as shown in Fig. S10a–c, where a pronounced negative-capacitance peak appears at ∼10 V under a slow sweep rate (0.005 V/s) but diminishes at higher sweep rates, consistent with prior reports [39]. Benchmarking in Fig. 3d further shows that the extracted SS_min_ and *I*_ON/OFF_ ratios place our *κ*-Ga_2_O_3_-gated FeHEMT among the leading candidates for low-power neuromorphic applications [39–51].

Transfer characteristics of the device were measured over an ultrawide temperature range from −270 to 210°C, as shown in Fig. 3e. These results demonstrate that the device can maintain its performance over such an extreme temperature range. Furthermore, heavy-ion irradiation characterization was conducted to evaluate the radiation tolerance, a property critical to celestial applications. The device still exhibits clear field-effect transistor behavior after ^181^Ta ion irradiation under an average flux of 1 × 10^4^ cm^−2^ s^−1^ and a total fluence of 1 × 10^7^ cm^−2^. The transfer characteristics show only a slight shift, the leakage current remains within the same order of magnitude, and the maximum mobility exhibits a moderate reduction of <11%, as shown in Fig. 3f and Fig. S11. These results support the robustness of the *κ*-Ga_2_O_3_-gated FeHEMT under spatial harsh-environment conditions.

### Emulations of reconfigurable memory behavior

**Figure 4.**
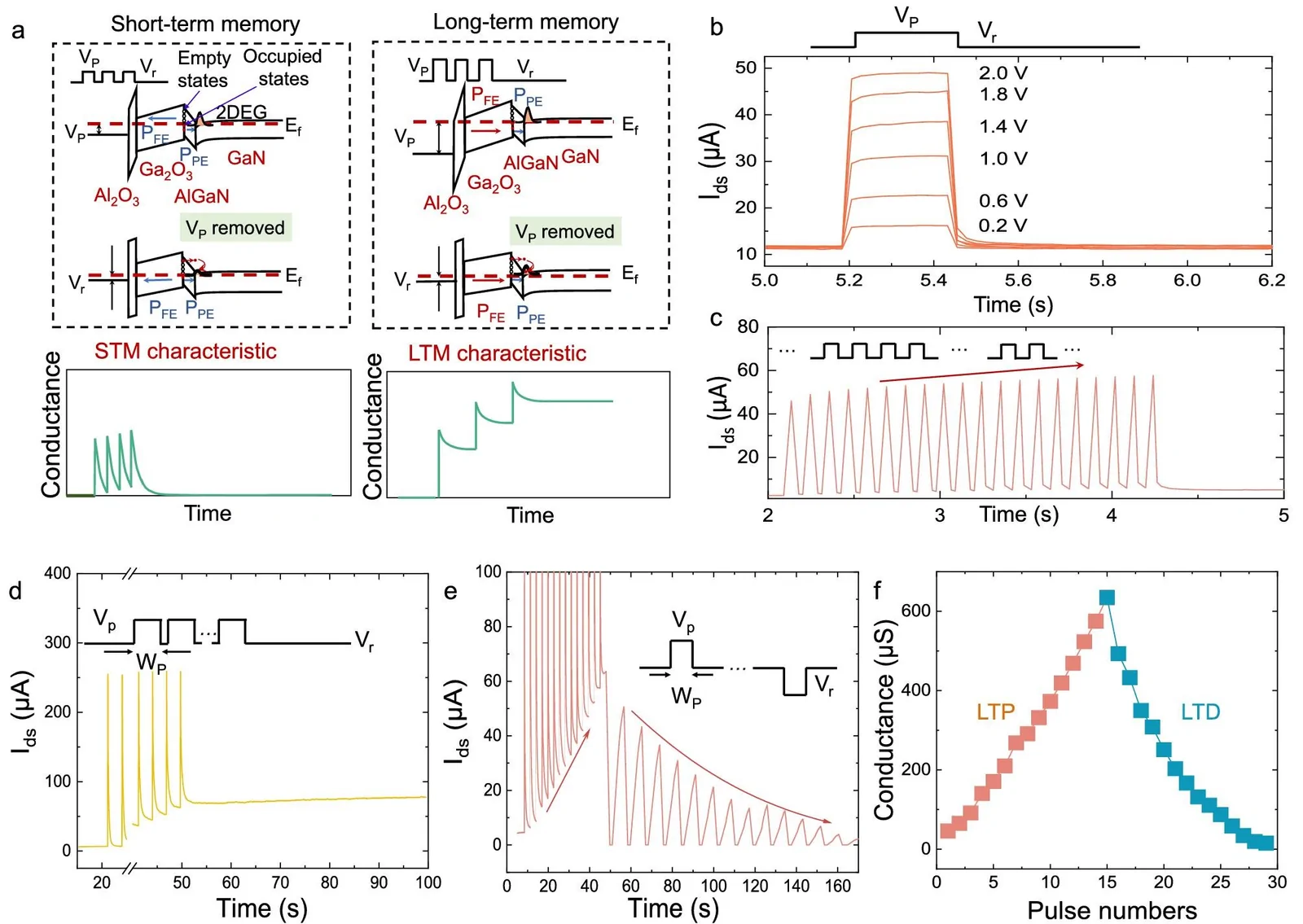
Ferroelectric plasticity in *κ*-Ga_2_O_3_-gated FeHEMT. (a) Band diagrams illustrating STM and LTM behaviors. (b) EPSC responses under gate voltage pulse from 0 to 2.0 V with a pulse duration of 200 ms, collected from the drain while the input pulses were applied to the gate. (c) Cumulative EPSC responses under sequential pulses with a time interval of 200 ms. (d) Retention of conductance states programmed by 20 V consecutive pulses. (e) Pulse-tunable programming and erasing of conductance using positive and negative gate pulses, respectively. (f) LTP and LTD induced by sequential gate pulses.

*κ*-Ga_2_O_3_ exhibits switchable ferroelectric polarization dynamics that inherently enable memory behaviors in two timing characteristics, thereby emulating both STM and LTM behaviors in a single device [23,52]. Figure 4a shows the operational band diagrams of STM and LTM in the *κ*-Ga_2_O_3_-gated FeHEMT. Under low pulsed gate voltage (*V*_p_ ≤ 5.0 V), where the electric field is below the coercive field, the ferroelectric polarization in *κ*-Ga_2_O_3_ remains unswitched. When *V*_p_ is applied, the enhanced gate field facilitates 2DEG accumulation at the AlGaN/GaN interface and increase channel conductance. Once *V*_p_ is removed, the 2DEG channel is gradually depleted, resulting in a transient decay in conductance. Concurrently, donor-like states at the *κ*-Ga_2_O_3_/AlGaN interface are emptied, contributing to partial restoration of 2DEG density and thereby enhancing the excitatory postsynaptic current (EPSC). As pulse numbers increases, the density of available donor-like interface states increases, progressively amplifying the EPSC and producing STM behavior (left panel of Fig. 4a). The cycling stability of the STM response under repeated pulse operation indicates that STM is unlikely to arise from polarization-state disturbance, as discussed in Fig. S12. In contrast, when high-amplitude pulses (*V*_p_ >20 V) are applied, the electric field exceeds the coercive field, triggering full ferroelectric polarization switching in *κ*-Ga_2_O_3_. The resulting strong polarization field enhances 2DEG confinement at the AlGaN/GaN interface even after pulse removal, enabling long-term retention characteristic of LTM operation (right panel of Fig. 4a).

STM behavior was experimentally mimicked by applying pulses with different amplitudes from 0 to 2.0 V and a fixed duration of 200 ms, as shown in Fig. 4b. The resulting channel current, recorded at a read voltage (*V*_r_ = 0.1 V), decays rapidly to baseline after *V*_p_ removal, analogous to a postsynaptic current decay. The decay kinetics are tunable via pulse amplitude, emulating the strength of presynaptic stimuli and governing the transient memory function. Sequential EPSCs are generated by low-amplitude pulses with a 200 ms intervals, as shown in Fig. 4c, exhibit progressive attenuation of baseline current along with increasing spike currents in the *κ*-Ga_2_O_3_-gated FeHEMT, confirming STM characteristic. Paired-pulse facilitation (PPF) was also demonstrated, with the PPF index reaching 114% at a 25 ms interval and decreasing to 104% at 300 ms, as shown in Fig. S13.

LTM behavior was demonstrated in Fig. 4d using six continuous gate pulses with a fixed pulsed width (*W*_p_ = 50 ms) and amplitude (*V*_p_ = 20 V). Since the applied field exceeds the coercive field, the device exhibits stable long-term characteristics. Figure S14 highlights precise modulation of multilevel conductance achieved by varying pulse numbers. Long-term potentiation and depression (LTP and LTD) were further achieved through 14 positive pulses (*W*_p_ = 50 ms, *V*_p_ = 20 V) and 14 negative pulses (*W*_p_ = 50 ms, *V*_p_ = −14 V), respectively (Fig. 4e). The extracted multilevel conductance states are summarized in Fig. 4f, demonstrating good linearity and reliable synaptic weight modulation characteristics. The stability and reproducibility of the programmed conductance states were further confirmed by repeated LTP/LTD measurements with different pulse numbers (Figs S15 and S16, respectively). The conductance evolves progressively with increasing pulse number and the repeated measurements show consistent dynamic responses. Additionally, the programmed LTP/LTD conductance states remain stable under temperature of 125°C, as shown in Fig. S17. Retention tests also demonstrate that distinguishable resistance states can persist over 100 s, as shown in Fig. S18.

### Celestial activity recognition and classification

**Figure 5.**
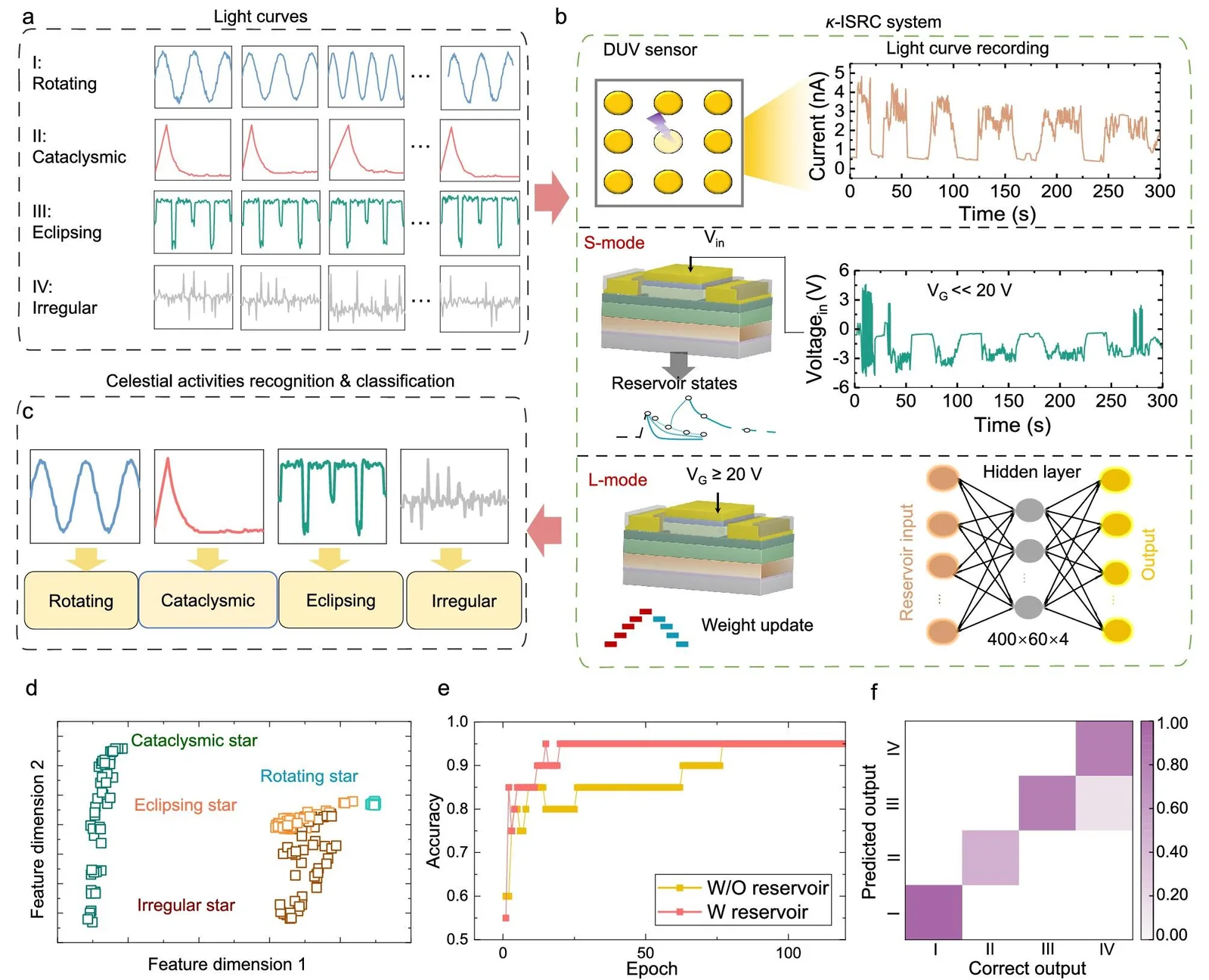
Light curve classification using a *κ*-ISRC system. (a) Measured light curves from the *κ*-Ga_2_O_3_ DUV sensor, representing four celestial activities. (b) Workflow of the light curve recognition task: measured light curves were obtained under 261 nm illumination, reservoir states were collected, and classification was performed through synaptic weight training in the FeHEMT. (c) Classification outputs for four celestial activities. (d) Dimensionality reduction of reservoir outputs using LDA. Each dot corresponds to an input, color-coded by class, showing clustered patterns. (e) Training accuracy of systems with and without the reservoir layer. (f) Confusion matrix for classifying four celestial activities, dominated by diagonal elements.

We assessed the capability of the developed *κ*-ISRC system for celestial activity recognition using the Optical Gravitational Lensing Experiment (OGLE) dataset [53], which contains four representative categories of astronomical phenomena-rotating, cataclysmic, eclipsing, and irregular (Fig. 5a). Each class includes 50 samples, with 90% used for training and 10% for testing. Figure 5b shows a workflow during the light curve recognition task. As shown in top panel of Fig. 5b, eclipsing binary system exhibits periodic intensity variations in light intensity as a dimmer companion partially obscures the primary star. Such temporal variations in luminosity are captured by the DUV sensor and translated into conductance modulations that reproduce the measured light curves. Under 261 nm illumination, the sensor exhibited a peak photocurrent of ∼5 nA, more than four orders of magnitude above the dark current (∼0.5 pA), confirming excellent DUV sensitivity (Fig. S19b). The reservoir states are obtained through simulations, in which the device parameters are extracted based on transient current response to different amplitudes. As shown in middle panel of Fig. 5b, the detected signals were subsequently mapped linearly to the input voltage range and projected into a high-dimensional space through multiplication using a random mask matrix of length ML, creating rich reservoir states through N parallel *κ*-Ga_2_O_3_-gated FeHEMTs in S-mode. Mapping *V*_G_ from 0 to 1 V generated 100 reservoir states (*ML* = 2, *N* = 2), which were then processed by a three-layer neural network of FeHEMTs in L-mode (400 input, 60 hidden, 4 output nodes; Fig. 5b). During training, synaptic weights (*W*_out_) are optimized using a backpropagation algorithm with a learning rate 0.001, and classification is achieved via a winner-take-all strategy. The resulting classification performance is summarized in Fig. 5c, where the four categories of celestial activity are clearly separated.

These output currents are then linearly mapped to the target input voltage range. Subsequently, the measured light curves are projected into a high-dimensional space by multiplication with a random mask matrix of length ML, generating interconnected virtual nodes in the time domain. The transformed signal is then applied to the reservoir layer of the *κ*-ISRC system, and the reservoir states are collected. The richness of reservoir states is achieved through N parallel *κ*-Ga_2_O_3_-gated FeHEMTs operating in S-mode. Specifically, light curves are mapped to *V*_G_ within a range from 0 to 1 V, generating 100 reservoir states (*ML* = 2 and *N* = 2), thereby enabling nonlinear projection of temporal inputs into a high-dimensional space. These reservoir states are subsequently collected and fed into the three-layer neural network consisted of *κ*-Ga_2_O_3_-gated FeHEMT in L-mode, for label inference. The input voltages are mapped to a range of 20–22 V. Such a neural network comprises 400 input nodes, 60 hidden nodes, and 4 output nodes, as shown in Fig. 5b. During the training process, the two-dimensional distribution of reservoir encodings obtained by linear discriminant analysis (LDA) (Fig. 5d) further demonstrates the discriminative capability of the system. Accuracy comparisons over 120 training epochs for systems (Fig. 5e) show that both reservoir-enabled and control systems achieve ∼95%, but the reservoir system converges significantly faster. The confusion matrix (Fig. 5f) further confirms robust classification using the *κ*-Ga_2_O_3_-gated FeHEMTs reservoir, with dominant diagonal elements indicating strong agreement between predictions and true labels. These results highlight the effectiveness of *κ*-ISRC for temporal pattern recognition in celestial activity classification.

Furthermore, stochastic noise analysis was performed to evaluate the tolerance of the system to device non-idealities such as cycle-to-cycle variation and read noise. The classification accuracy remains at a high level, exceeding 90% even at a noise level of 50%, which confirms the noise-robustness of the FeHEMT-based reservoirs (Fig. S20a and b).

## CONCLUSION

In summary, we have demonstrated *κ*-Ga_2_O_3_-gated FeHEMT that integrate ferroelectric functionality with wide-bandgap heterostructures, achieving sub-60 mV/dec switching, high ON/OFF current ratios, and polarization-programmable states. The negative capacitance effect of the Al_2_O_3_/*κ*-Ga_2_O_3_ stack enables steep-slope operation beyond the Boltzmann limit, while the inherent radiation hardness of *κ*-Ga_2_O_3_ ensures stable performance under harsh environments. By leveraging these unique device characteristics, we further developed a *κ*-ISRC framework for celestial activity recognition. The device intrinsically performs nonlinear transformation and memory processing, enabling compact, energy-efficient classification of solar flares, cosmic-ray bursts, and pulsar emissions with an accuracy of ∼95%.

This work establishes *κ*-Ga_2_O_3_ as a multifunctional material that not only extends the frontier of ferroelectric-gated GaN transistors but also provides a radiation-tolerant platform for intelligent sensing and low-power neuromorphic computation. Notably, by unifying sensing, processing, and memory within a single material system, the *κ*-ISRC minimizes data transfer, improves computational efficiency, and ensures resilience in harsh conditions, eliminating the bonding and interconnect complexity common in heterogeneous reservoir computing systems [14,54–58]. Furthermore, the *κ*-Ga_2_O_3_-gated FeHEMT has demonstrated exceptional radiation resistance and ultrawide operating temperature range. The comparative analysis with other material systems is summarized in Table S1. This intrinsic resistance under irradiation renders it highly suitable for deployment in harsh environments and mission-critical applications. The demonstrated synergy between steep-slope device physics and in-sensor computing architectures opens new pathways toward integrated electronics for next-generation aerospace and deep-space applications.

## METHODS

### Material growth and device fabrication

The *κ*-Ga_2_O_3_-gated FeHEMT was fabricated on an AlGaN/GaN heterostructure grown on a SiC (0001) semi-insulating substrate. The epitaxial stack, deposited by metal organic chemical vapor deposition, comprises an AlN nucleation layer, a GaN buffer layer (∼2 μm), a 300 nm unintentionally doped GaN (UID-GaN) channel layer, and a 25 nm Al_0.25_Ga_0.75_ N barrier layer. The fabrication flow is illustrated in Fig. S1.

Ohmic contacts were formed by depositing a Ti/Al/Ni/Au metal stack via electron-beam evaporation, followed by rapid thermal annealing at 840°C for 60 s in N_2_ ambient. Mesa isolation was defined by inductively coupled plasma (ICP) etching using a BCl_3_/Cl_2_ gas mixture. A SiN_*x*_ passivation layer was then deposited by plasma-enhanced chemical vapor deposition from SiH_4_ and NH_3_ precursors. The gate region was patterned by photolithography. Subsequently, a 50 nm *κ*-Ga_2_O_3_ ferroelectric layer was epitaxially grown by mist chemical vapor deposition (mist-CVD) at 710°C, using GaCl_3_ dissolved in deionized water or alcohol-based solvents as the precursor. A 10 nm Al_2_O_3_ dielectric was deposited by atomic layer deposition (ALD) at 300°C using trimethylaluminum (TMA) and H_2_O precursors, and the Al_2_O_3_/*κ*-Ga_2_O_3_ stack was patterned by ICP. Finally, a Ni/Au gate electrode was deposited by e-beam evaporation, and the 100 nm SiN_*x*_ in the source/drain access regions was removed by reactive ion etching (RIE).

### Celestial activity recognition simulations

The key functions of the *κ*-Ga_2_O_3_-based DUV sensor and FeHEMT were characterized by experiment, and the recognition task was based on simulation. The temporal light-curve signals were first normalized and linearly mapped to the input voltages. A random mask matrix was used to generate time-multiplexed input signals. The masked input sequences were applied to model-based FeHEMT operating in the S-mode for reservoir state generation. Finally, the reservoir states were subsequently processed by a model-based FeHEMT readout layer operating in the L-mode for label inference.

### Device and materials characterizations

Electrical characterization was performed using Keithley 2636A and 4200-SCS. Surface morphology was analyzed by atomic force microscopy (AFM, SPA-400, Seiko Instruments Inc.) operated with an SPI-4000 probe station. Ferroelectric response was probed by PFM using a Cypher AFM system (Asylum Research, Oxford Instruments) with Nanosensors PPP-EFM cantilevers (Cr/PtIr-coated Si tips, nominal radius ∼25 nm). Structural characterization was conducted using a Gemini 500 STEM. Cross-sectional samples of the Ni/Au/Al_2_O_3_/*κ*-Ga_2_O_3_/AlGaN/GaN stack was prepared by focused ion beam (FIB, Helios 600). The layered structure and elemental distribution were further examined by STEM combined with energy-dispersive X-ray spectroscopy (EDS, Tecnai F20).

### Theoretical calculations

Initial crystal structures were derived from experimental X-ray diffraction refinements [59]. AIMD simulations based on density functional theory [60], were performed with the Quickstep/CP2K code [61]. Calculations employed DZVP-MOLOPT-SR-GTH basis sets and Goedecker–Teter–Hutter (GTH) pseudopotentials [62], with a 2 × 2 × 2 supercell of *κ*-Ga_2_O_3_ (320 atoms) and a 400 Ry energy cutoff. To obtain a thermally stable structure, the supercell was equilibrated in the NVT (Number of particles, Volume, Temperature) ensemble at 300 K using a Nosé–Hoover thermostat [63,64]. Subsequently, a constant electric field of 0.005 a.u. (0.26 V/nm) was applied along the $[{00\bar{1}}]$ direction to mimic the influence of an external field [58].

## Supplementary Material

nwag362_Supplemental_File
