# Supplementary material for: Celestial neuromorphics based on ferroelectric gallium oxide
Source: Natl Sci Rev. 2026 Jun 12;13(15):nwag362. doi: 10.1093/nsr/nwag362 (PMC13547606; doi:10.1093/nsr/nwag362)
Supplement: Supplementary file 1 [file nwag362_supplemental_file.pdf]

Supplementary information for

Celestial neuromorphics based on ferroelectric gallium oxide

Ke Xu[1,†], Zhannan Guan[1,†], Mengjiao Pei[1,2,†], Yurong Luo[1], Yonghao Zhu[3], Xinxin Yu[4], Lin Hao[1], Songhao Gu[1], Jiahao Yao[1], Zhanhua Li[1], Xinyi Pei[1], Yuhao Zhang[5], Han Wang[5], Changjin Wan[1,6]*, Qing Wan[6], Rong Zhang[1], Jiandong Ye[1]**

[1]School of Electronic Science and Engineering, National Laboratory of Solid-State Microstructures, Nanjing University, Nanjing 210023, China.

[2] Department of Applied Physics, The Hong Kong Polytechnic University, Hong Kong, China

[3] State Key Laboratory of Superlattices and Microstructures, Institute of Semiconductors, Chinese Academy of Sciences, Beijing 100083, China.

[4] CETC Key Laboratory of Carbon-based Electronics, Nanjing Electronic Devices Institute, Nanjing 210016, China.

[5] Department of Electrical and Electronic Engineering, University of Hong Kong, Hong Kong 999077, China

[6] Yongjiang Laboratory (Y-LAB), Ningbo 315202, China.

Supplementary section 1: Computational details for the energy-polarization (E-P) characteristic of κ-Ga_2O_3

We investigated the Energy-Polarization (E-P) characteristic of 2 × 2 × 2 supercell of κ-Ga_2O_3 during polarization switching using nudged elastic band (NEB) simulations [1] through VASP. The electric polarization of κ-Ga_2O_3 was calculated using the Berry phase method [2].

Ferroelectric and reversed-ferroelectric states of κ-Ga_2O_3 served as the two variants (±P); both structures were fully relaxed prior to the NEB calculations. The paraelectric phase (i.e. the non-polar reference state with P≈0) by evenly interpolating between the ferroelectric and reversed ferroelectric configurations and selecting the midpoint along the interpolation path. During NEB relaxations, all atomic coordinates were fully optimized with the lattice vectors constrained to the zero-stress ferroelectric cell (volume effects are small over the flipping path). A plane-wave cutoff of the calculation was set to 520 eV, spring constants of 5 eV/Å^2 were used to maintain even image spacing. A 5 × 3 × 3 Monkhorst-Pack k-point mesh was used for calculation. Energy minimization and ionic relaxation were performed with a tolerance of 10^{-6} eV per formula unit for the total energy, and 0.05 eV/Å on each nuclear for the atomic force, respectively.

Supplementary section 2: Calculation of electron mobility in the channel.

Figure S8a shows the capacitance-voltage (*C-V*) characteristics measured during a forward V_G sweep from -22 V to 20 V, followed by a reverse sweep from 20 V back to -22 V. The *C-V* trend aligns with the transfer characteristics and exhibits a counterclockwise hysteresis window. The gate control of the cannel current (I_{ds}) is quantified by the transconductance (g_m), defined as the partial derivative of the I_{ds} with respect to the V_G [3]:

$$g_m = \frac{\partial I_{ds}}{\partial V_G} \tag{S1}$$

As shown in **Fig. S8b**, after κ-Ga_2O_3 is poled downward, the overall g_m-V_G curve exhibits a negative shift compared to the poled-up state, consistent with the counterclockwise hysteresis observed in the transfer characteristics. Under the poling down condition, the peak transconductance reaches 395 mS/mm, higher than 293 mS/mm in the poling up state. This g_m variation indicates that the FeHEMT switching performance is superior during the negative V_{GS} sweep. In the subthreshold region, higher g_m reflects greater sensitivity of the I_{ds} to V_G, resulting in steeper transfer characteristic and improved switching performance under the poling down state.

Field-effect mobility of channel carriers is calculated using [4, 5]:

$$\mu = \frac{g_m V_{ds}}{C \frac{W_G}{L_G} (V_{DS} - R_t I_{ds})^2} \tag{S2}$$

$$R_t = R_{GS} + R_{GD} + R_{CD} + R_{CS} \tag{S3}$$

$$R_{GS} = R_{sh} \frac{L_{GS}}{W_G} \tag{S4}$$

$$R_{GD} = R_{sh}\frac{L_{GD}}{W_G} \tag{S5}$$

where μ is the field-effect mobility of channel carriers, g_m is the transconductance, C is the capacitance per unit area, W_G and L_G are the gate width and length, respectively, and R_t is the channel resistance outside the gate region; L_{GD} and L_{GS} denote the distances from the gate to the source and drain, respectively; R_{CS} and R_{CD} are the source and drain contact resistances, R_{sh} is the sheet resistance, and R_{GS} and R_{GD} are the gate-to-source/drain resistances, respectively.

The values of R_{sh}, R_{CS} and R_{CD} were measured using the transmission line method (TLM) [6], yielding 297, 17.1, 17.1 Ω, respectively. Using these parameters, the relationship between μ and V_G is plotted in **Fig. S8c**. When a positive V_G is applied, the polarization direction in κ-Ga_2O_3 aligns toward the AlGaN side, enhancing internal polarization and increasing the channel carrier mobility to 1550 $cm^2/V\cdot s$. Conversely, under negative V_G, the polarization of κ-Ga_2O_3 reverts upward, partially depleting electrons in the channel and degrading electron mobility to 1250 $cm^2/V\cdot s$.

Supplementary section 3: Influence of rotational domains on device uniformity

The presence of rotational domains in the κ-Ga_2O_3 epilayer may have implications for device-to-device uniformity, particularly when the material is implemented in array-level reservoir computing systems. Because orthorhombic κ-Ga_2O_3 is heteroepitaxially grown on a hexagonal template, rotational-domain boundaries such as twin or antiphase boundaries can be introduced during film growth. These boundaries may locally modify the defect/trap distribution and electrostatic environment, and therefore could act as preferential leakage paths or carrier scattering centers. In the κ-Ga_2O_3 FeHEMT, such local inhomogeneity may further affect the vertical electric field across the gate stack and the charge trapping/de-trapping behavior near the κ-Ga_2O_3 /AlGaN interface, potentially contributing to variations in threshold voltage and ferroelectric switching voltage. Since uniform conductance modulation and reproducible polarization switching are important for reservoir-state generation and synaptic weight programming, reducing rotational-domain density and improving epitaxial uniformity will be important directions for future large-scale κ-Ga_2O_3 FeHEMT arrays.

Supplementary section 4: Calculation of energy consumption per synaptic operation.

For the trained readout layer, the energy consumption was recalculated from the conductance states in **Fig. 4f**. The read current was estimated as:

$$I_a = V_{ds} \times G_a \tag{S6}$$

From the LTP/LTD conductance modulation, the device conductance ranges from approximately 15 to 620 μS, with an average conductance of about 250 μS. Under V_{DS}=5 V and a time step of 0.025 s, the energy per operation is estimated as:

$$E = V_{ds}^2 G_a \Delta t \approx 1.56 \times 10^{-4}\, J \tag{S7}$$

Therefore, the generation/readout of 100 reservoir states consumes approximately 15.6 mJ. We used 100 reservoir states as one action in our celestial activity recognition task. In the other word, the energy consumption for reservoir state collection is 15.6 mJ/action.

In addition, the total energy consumption for recognizing one input sample can be estimated by the reservoir state collection and the trained readout network for label inference:

$$E_{tot} = E_{reservoir} + E_{readout} = 15.6\, mJ + 0.97\, J = 0.9856\, J \tag{S8}$$

Thus, the overall energy consumption is approximately 985.6 mJ per input sample.

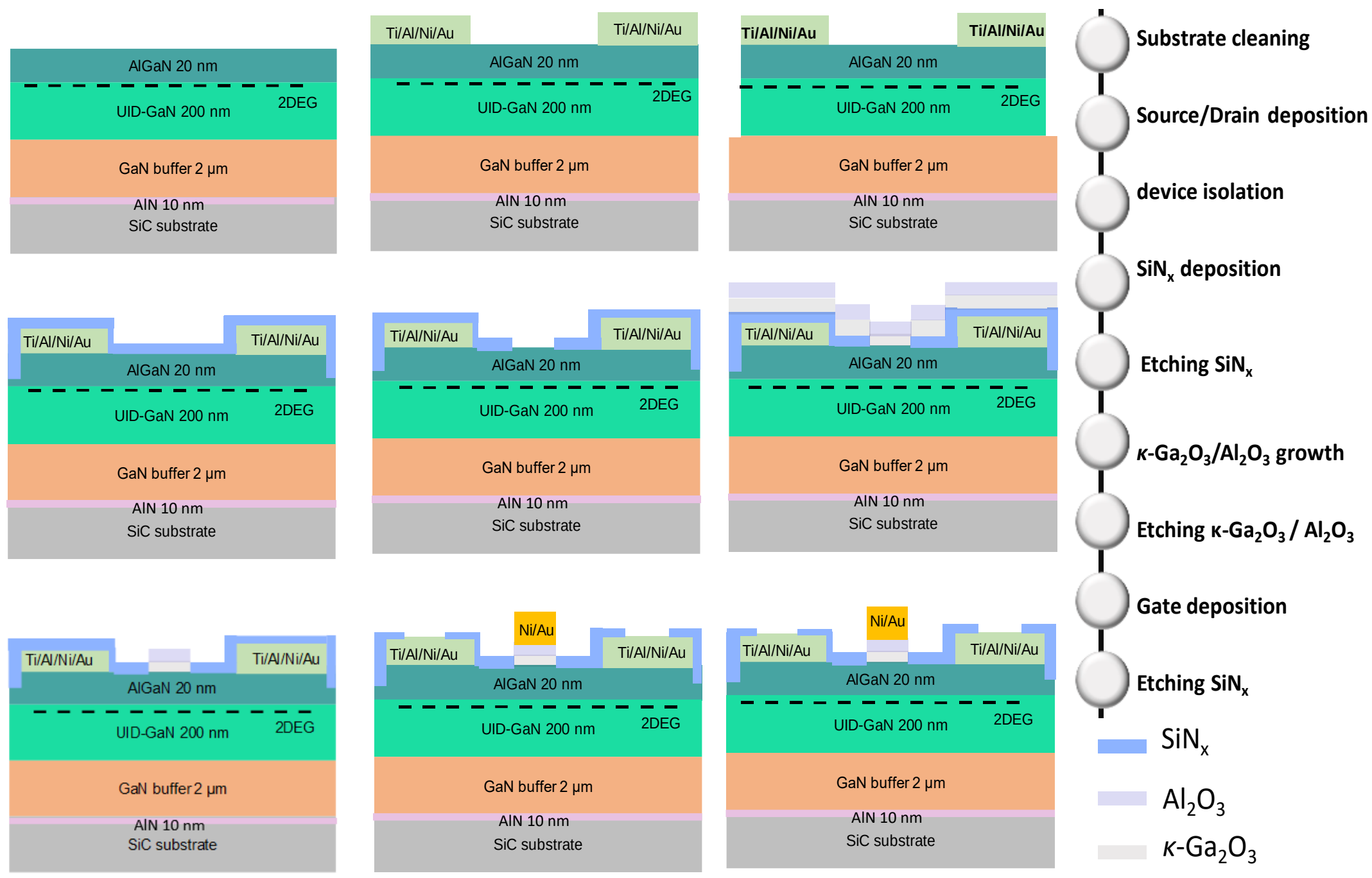

Supplementary Fig. S1. Fabrication process flow of κ-Ga_2O_3 gated FeHEMT

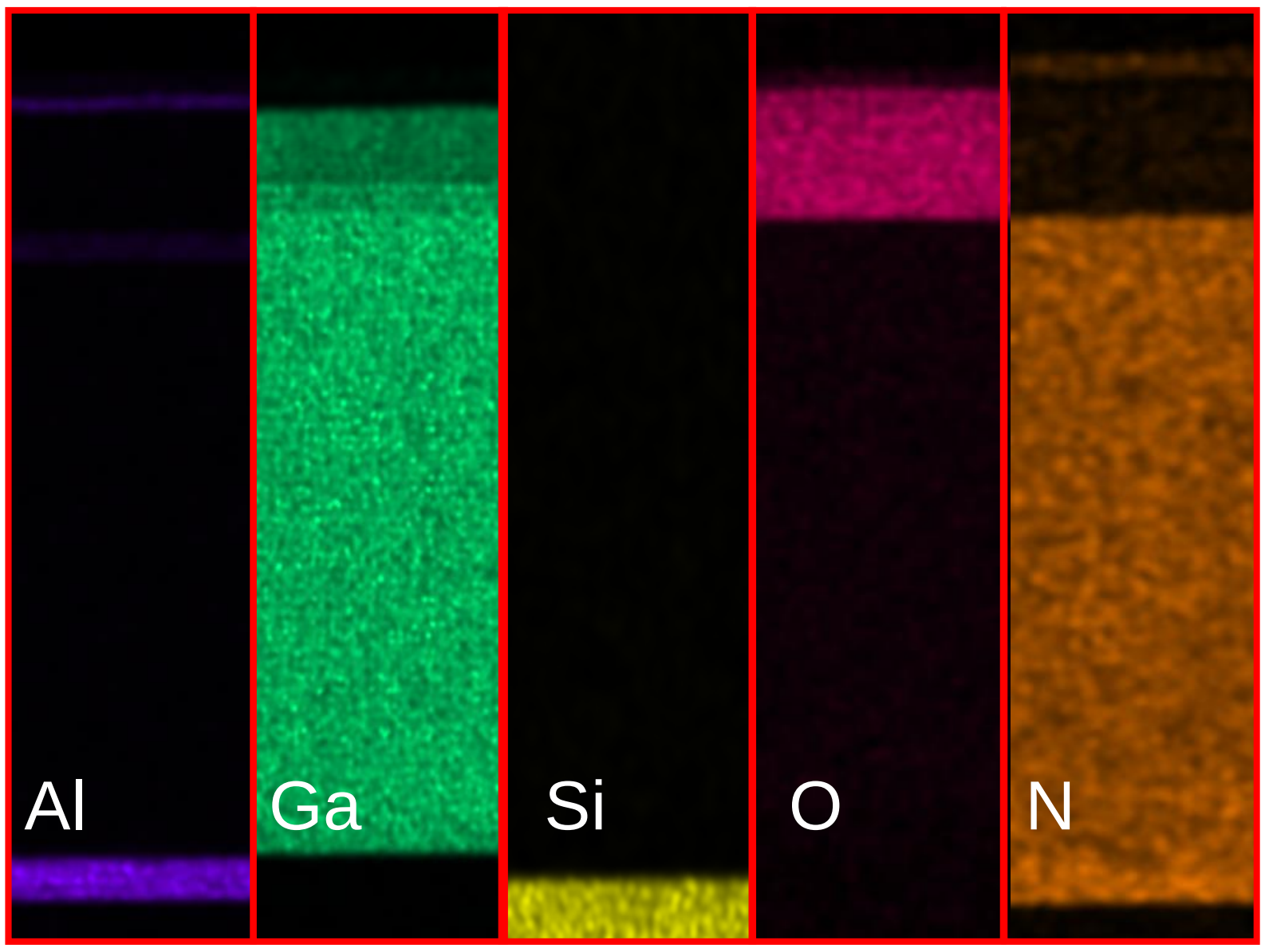

Supplementary Fig. S2. Cross-sectional element mapping of Al, Ga, Si, O and N from the transistor gate to channel obtained via energy dispersive X-ray spectroscopy (EDS).

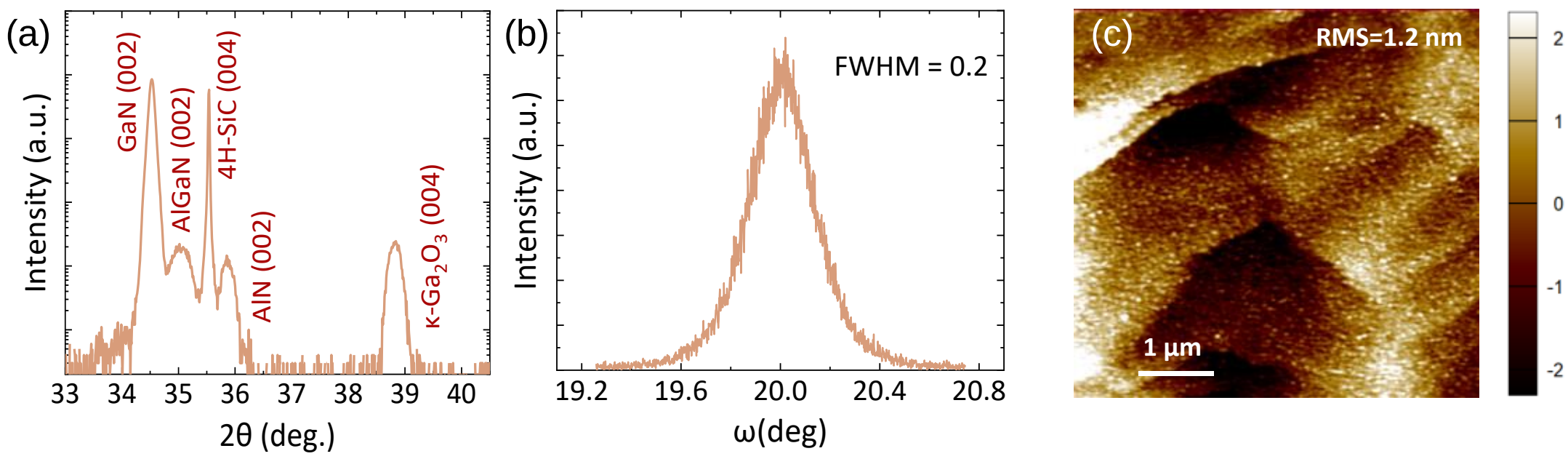

Supplementary Fig. S3. Structural and surface characterization of κ-Ga_2O_3 epilayers. **(**a) XRD 2θ pattern of κ-Ga_2O_3 epilayer on AlGaN/GaN heterostructure. (b) High-resolution XRD rocking curve with a FWHM of 0.2°. (c) AFM image showing surface morphology with root-mean-square (RMS) roughness of 1.2 nm.

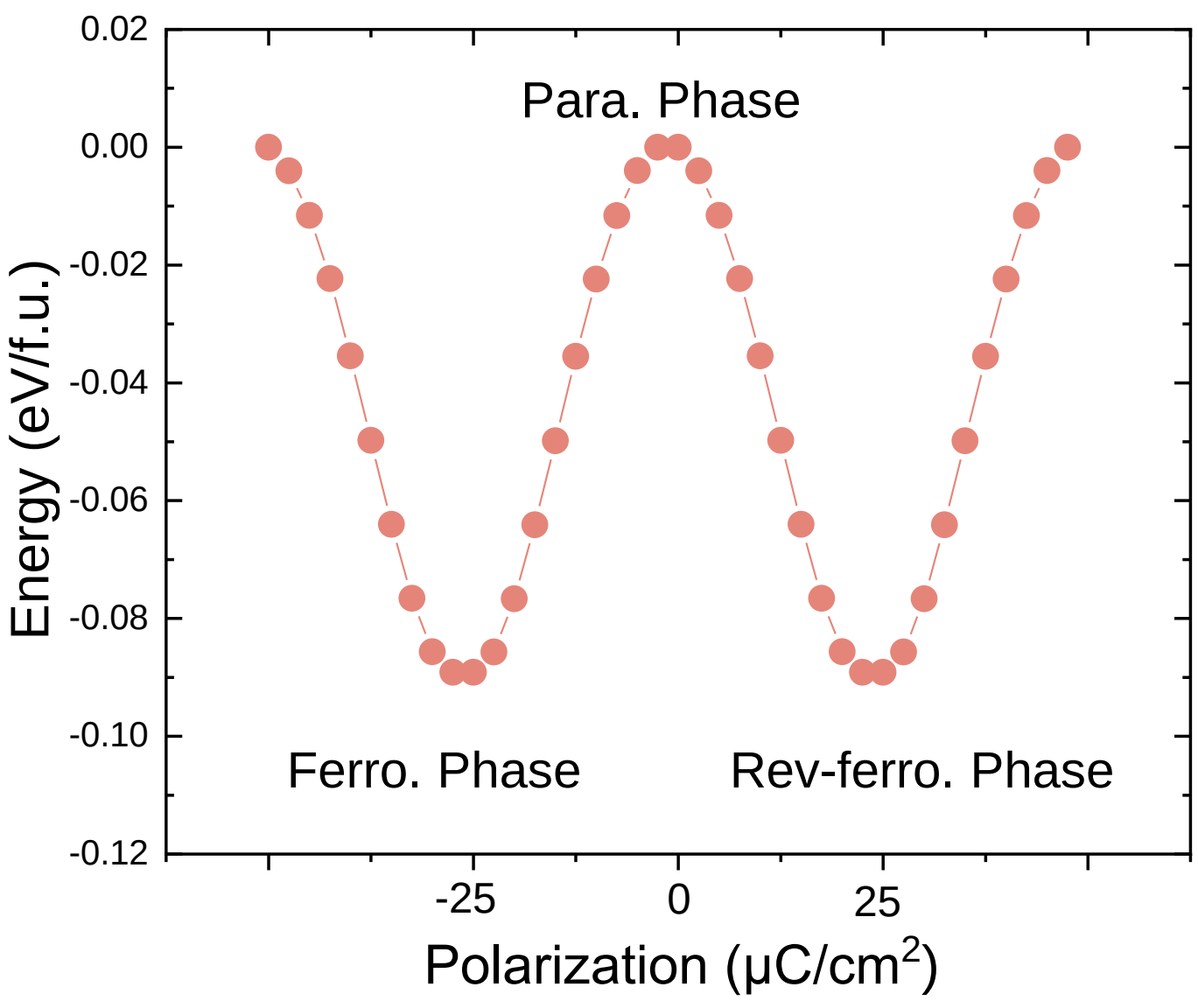

Supplementary Fig. 4. Energy-polarization curve of κ-Ga_2O_3 shows a characteristic double-well profile, indicating the spontaneous polarization characteristic. The corresponding polarization–electric field (P-E) curve includes a negative slope region, corresponding to the negative capacitance effect.

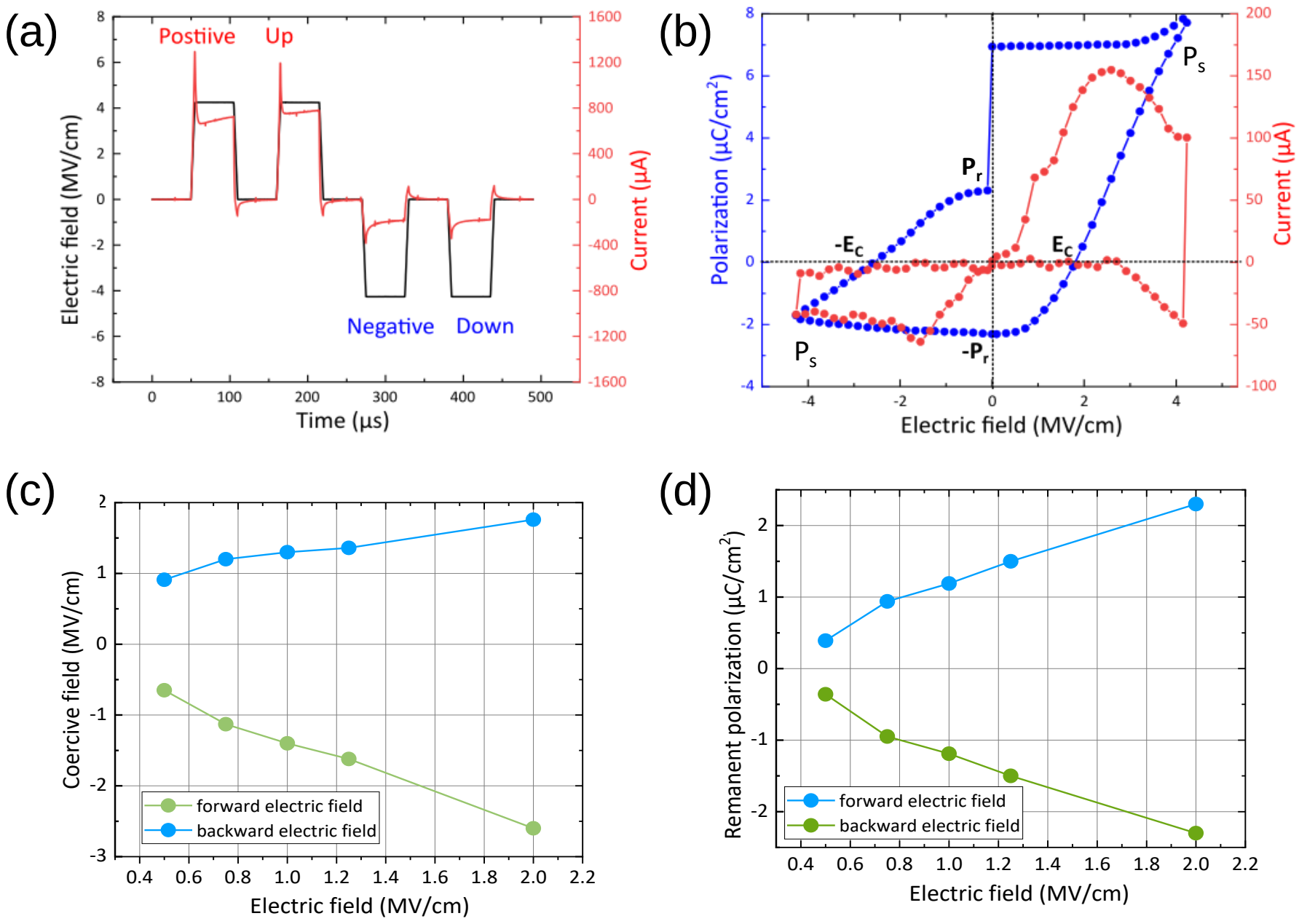

Supplementary Fig. 5. Ferroelectric characterization of κ-Ga_2O_3 epilayer. (a) Square-wave voltage pulses applied in PUND testing. (b) P-E and I-V results from PUND measurements. (c) Coercive field (E_C) versus applied electric field. (d) Remnant polarization (P_r) versus applied electric field in κ-Ga_2O_3.

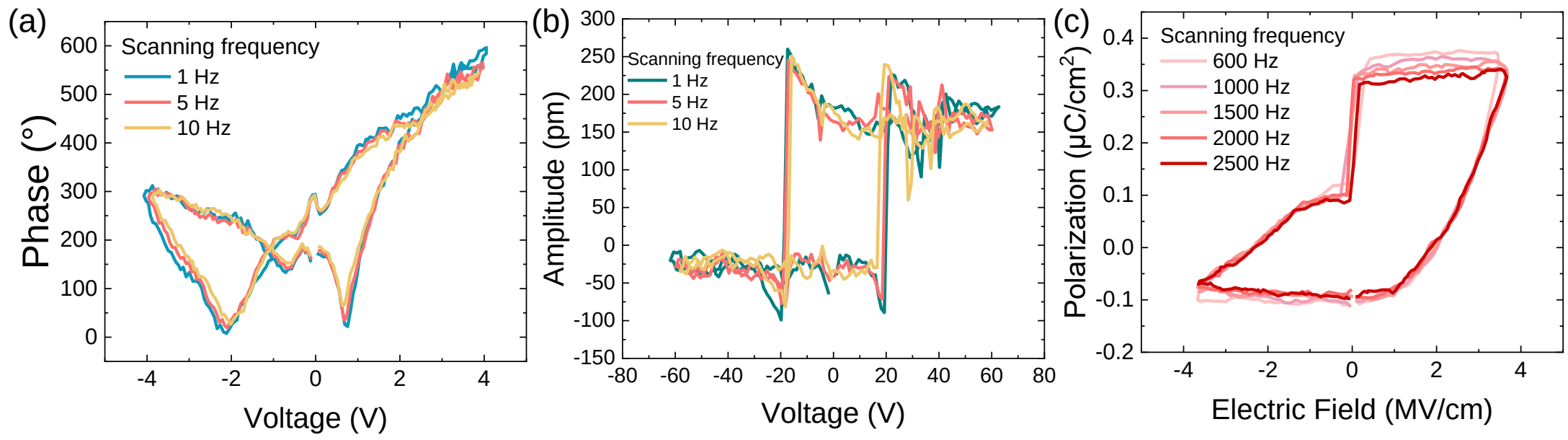

Supplementary Fig. 6. Frequency-dependent local and macroscopic ferroelectric switching characteristics of *κ*-Ga_2O_3. (a) Frequency-dependent PFM phase hysteresis loops. (b) Frequency-dependent PFM amplitude loops. (c) Frequency-dependent macroscopic P-E hysteresis loops.

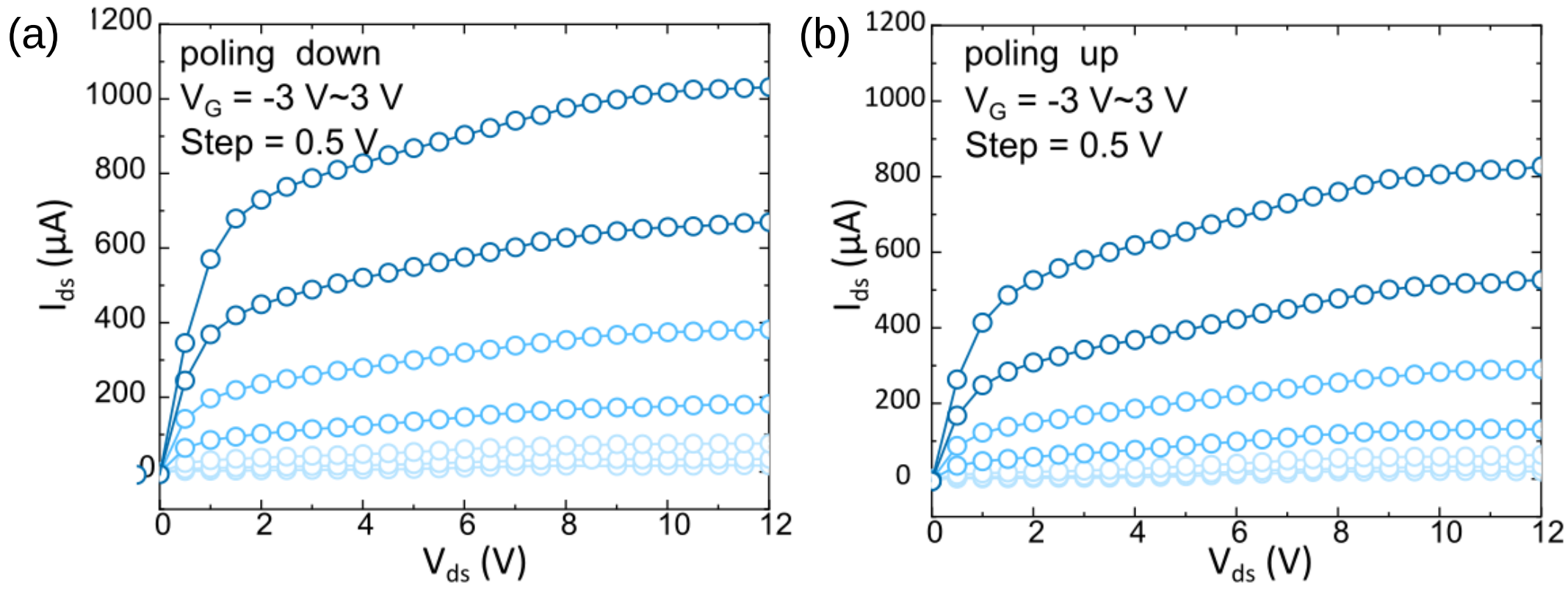

Supplementary Fig. 7. Output characteristics of κ-Ga_2O_3-gated FeHEMT under (a) poling down and (b) poling up states.

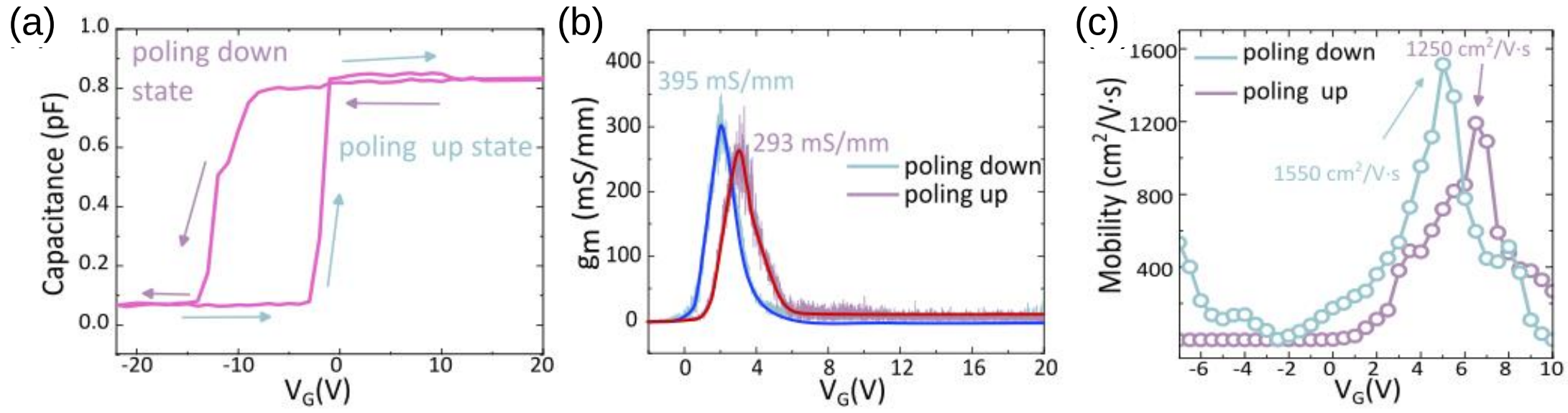

Supplementary Fig. 8. Capacitance (*C*), transconductance (g_m), and channel mobility (μ) characteristics of κ-Ga_2O_3 gated FeHEMT under different poling states. (a) *C-V* characteristics under poling down and poling up states. (b) g_m as a function of gate voltage (V_{GS}). (c) μ as a function of V_{GS}.

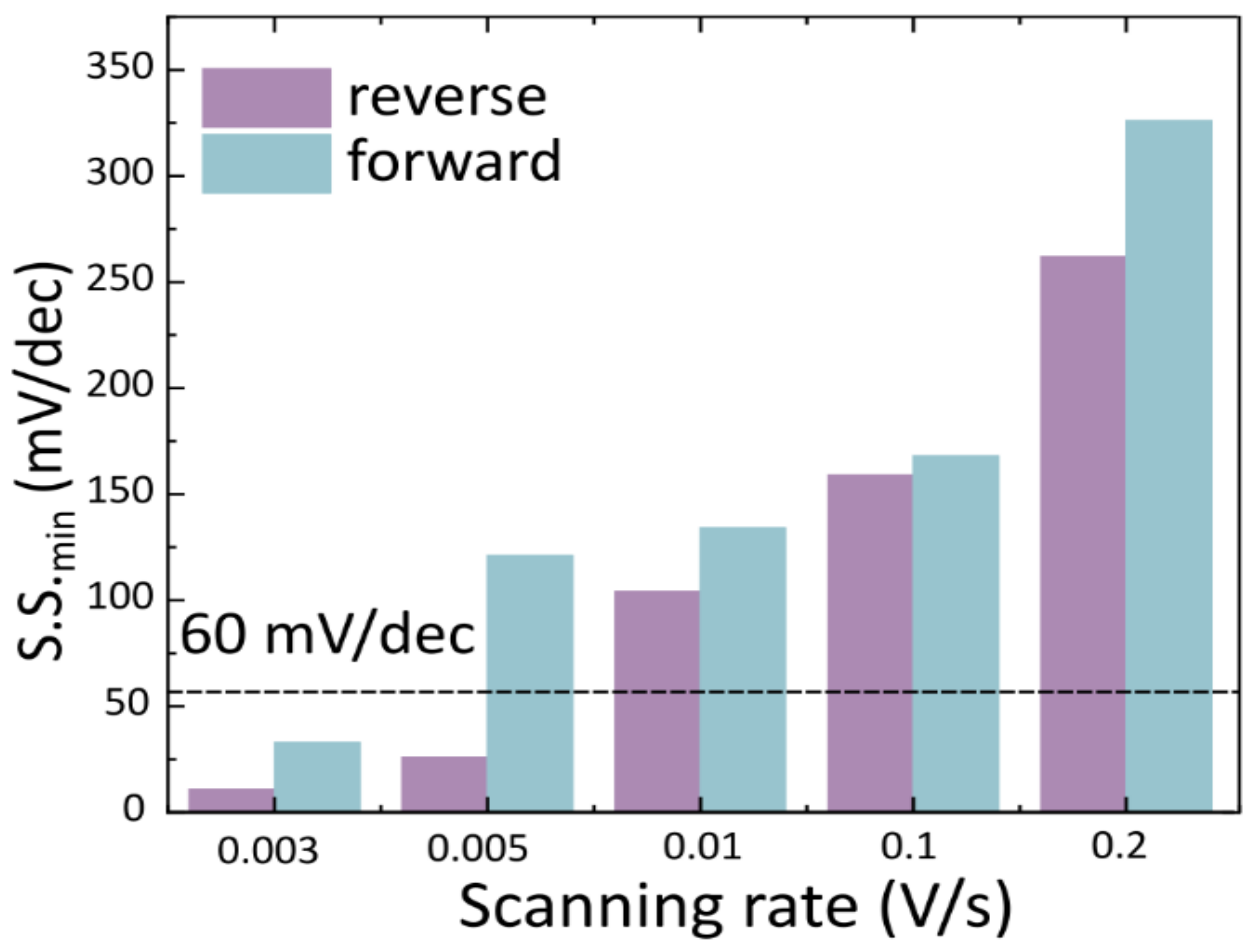

Supplementary Fig. 9. Minimum subthreshold swing (SS_{min}) measured under forward and backward gate voltage (V_G) sweeps at different scan rates.

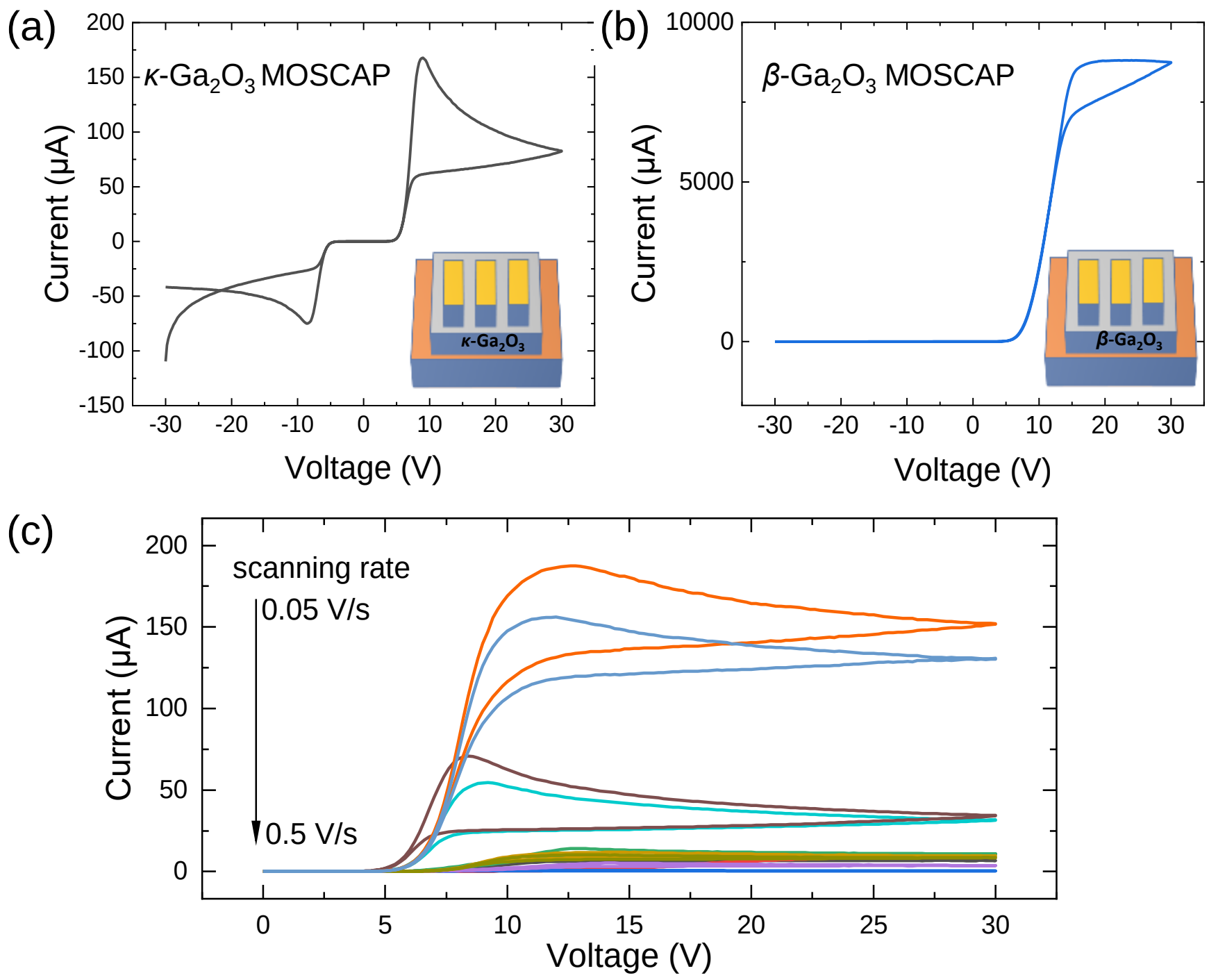

Supplementary Fig. 10. Hysteresis behavior of κ-Ga_2O_3 and β-Ga_2O_3 metal-oxide-semiconductor (MOS) capacitors. (a) *I-V* of κ-Ga_2O_3 MOS capacitor at a V_G sweep rate of 0.005 V/s. (b) *I-V* of β-Ga_2O_3 MOS capacitor at a sweep rate of 0.005 V/s. (c) *I-V* of κ-Ga_2O_3 MOS capacitor at sweep rates from 0.005 to 0.5 V/s.

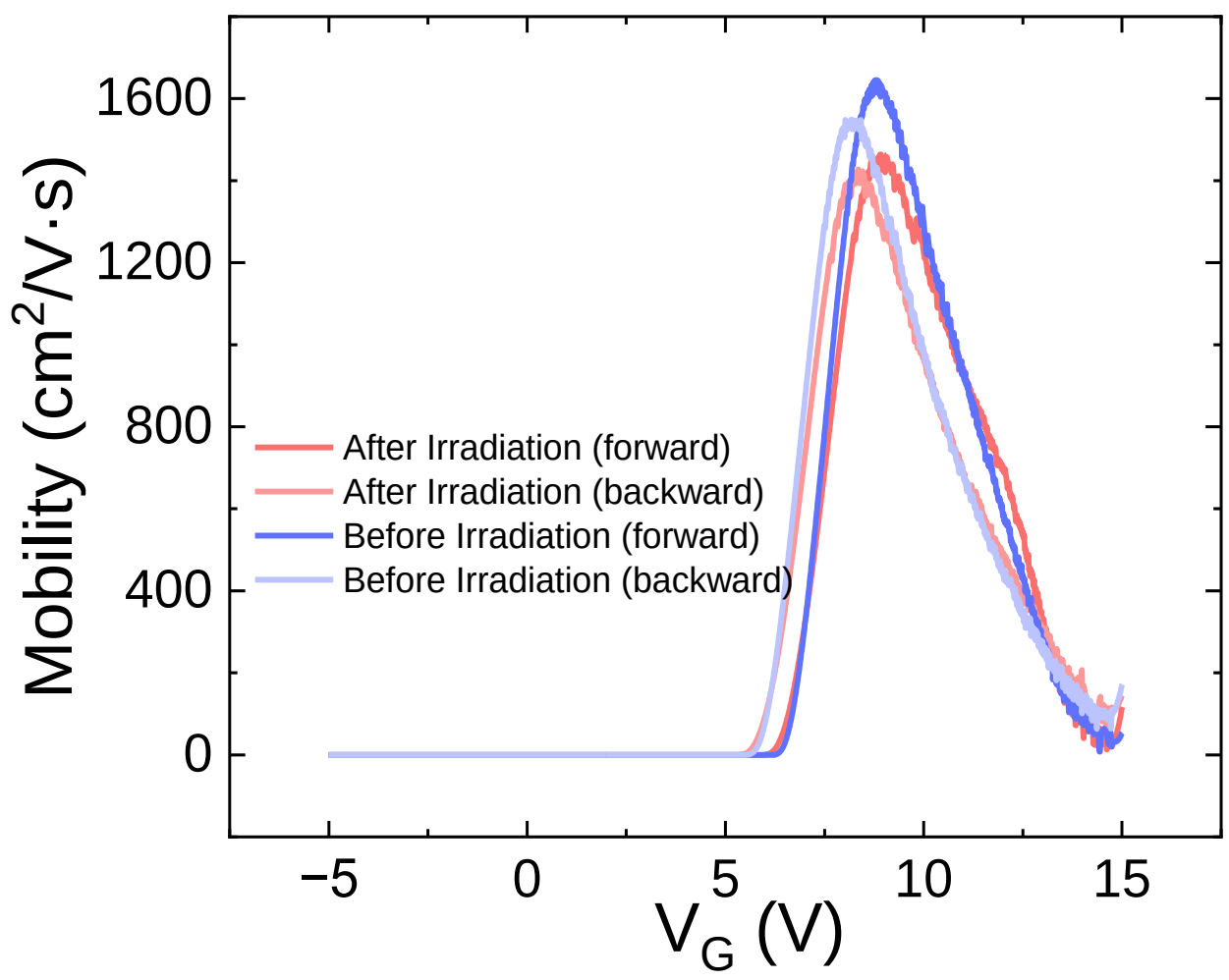

Supplementary Fig. 11. Extracted mobility of the *κ*-Ga_2O_3 FeHEMT before and after ^{181}Ta heavy-ion irradiation.

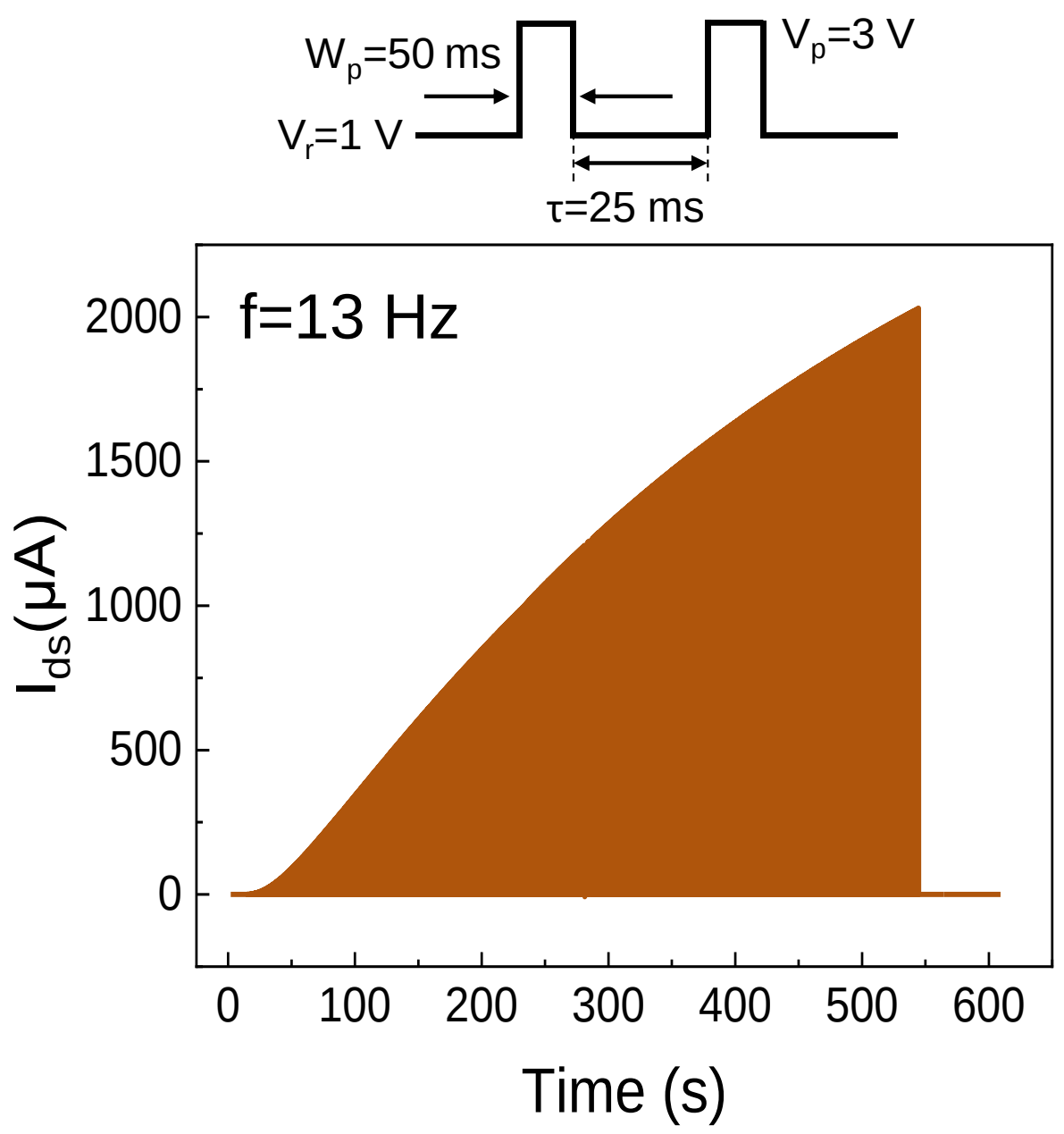

Supplementary Fig. 12. Cumulative STM current evolution under repeated gate stimulation with a 25 ms pulse interval.

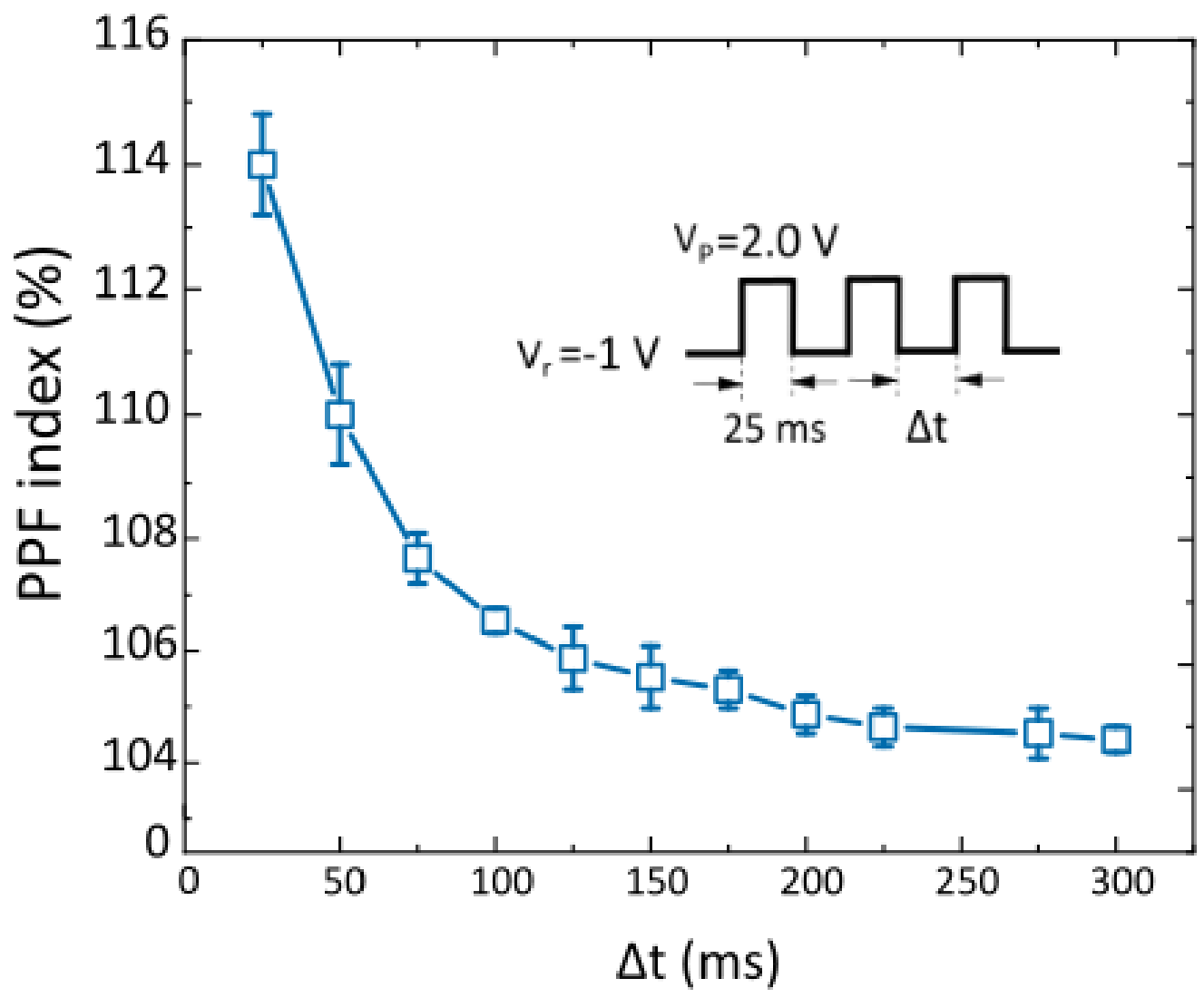

Supplementary Fig. 13. Paired-pulse facilitation (PPF) in the *κ*-Ga_2O_3-gated FeHEMT with pulse amplitude V_p of 2.0 V.

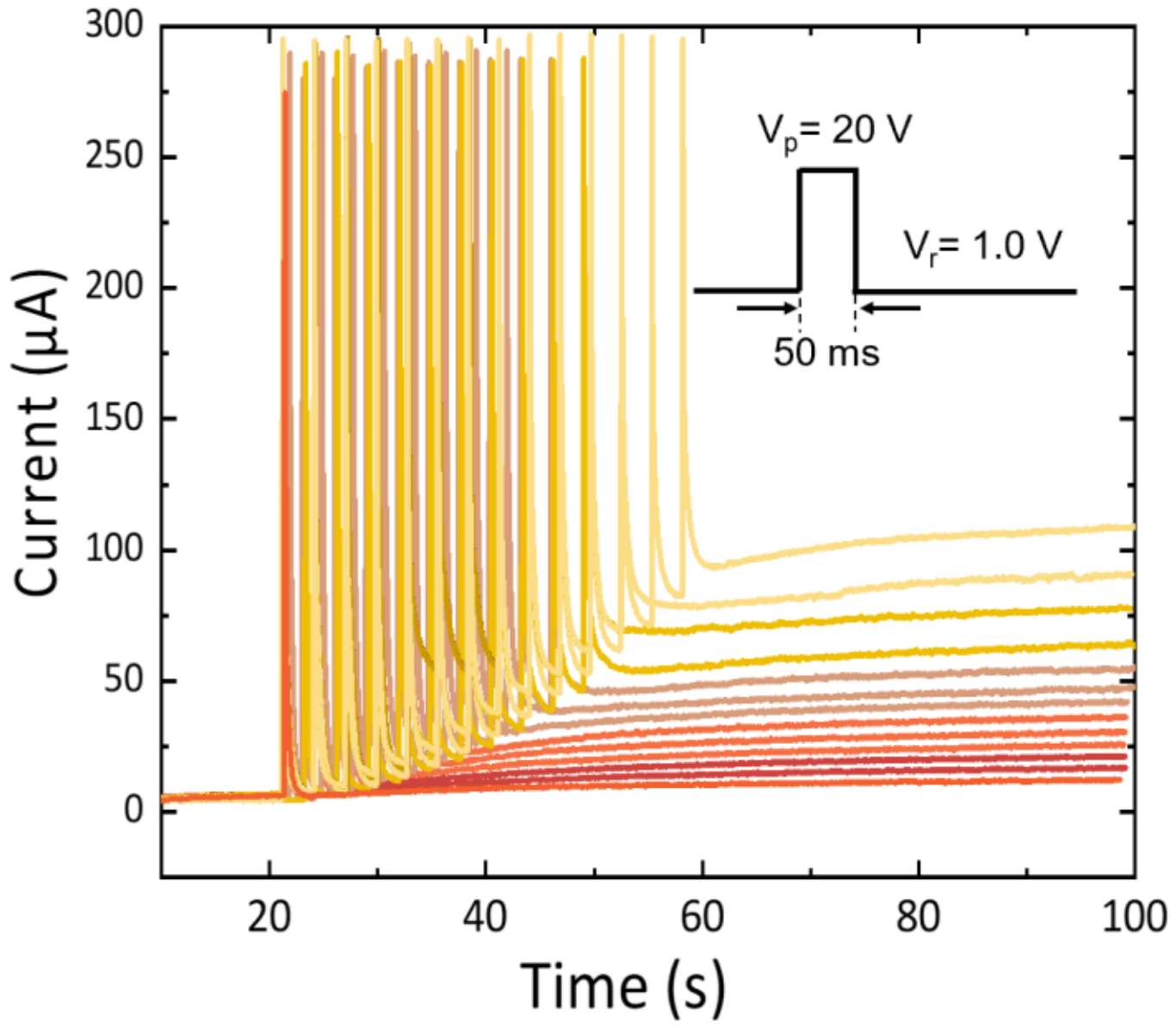

Supplementary Fig. 14. Multilevel nonvolatile current modulation of κ-Ga_2O_3-gated FeHEMT under repetitive programming pulses with V_p of 20 V.

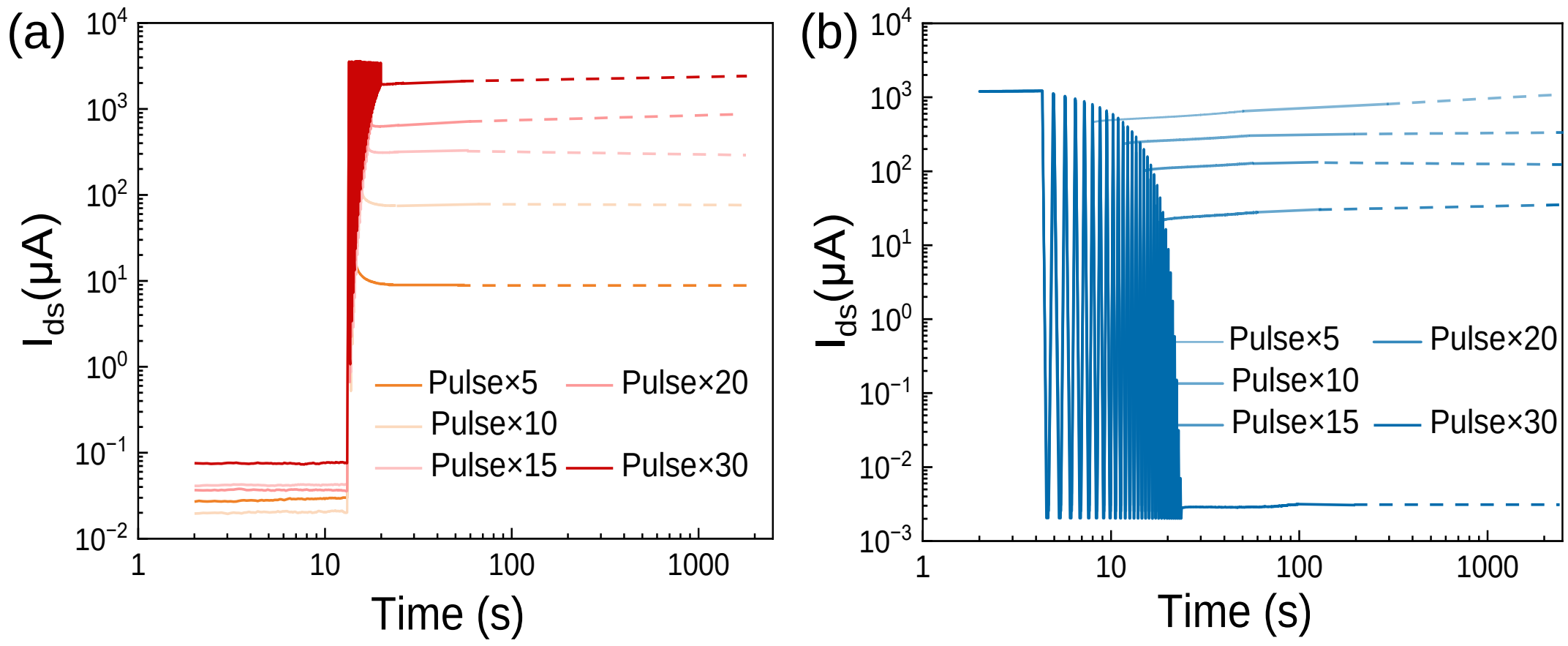

Supplementary Fig. 15. Long-Term Potentiation/Depression Characteristics of the *κ*-Ga_2O_3 FeHEMT. (a) Long-term potentiation under consecutive pulses. (b) Long-term depression under consecutive pulses

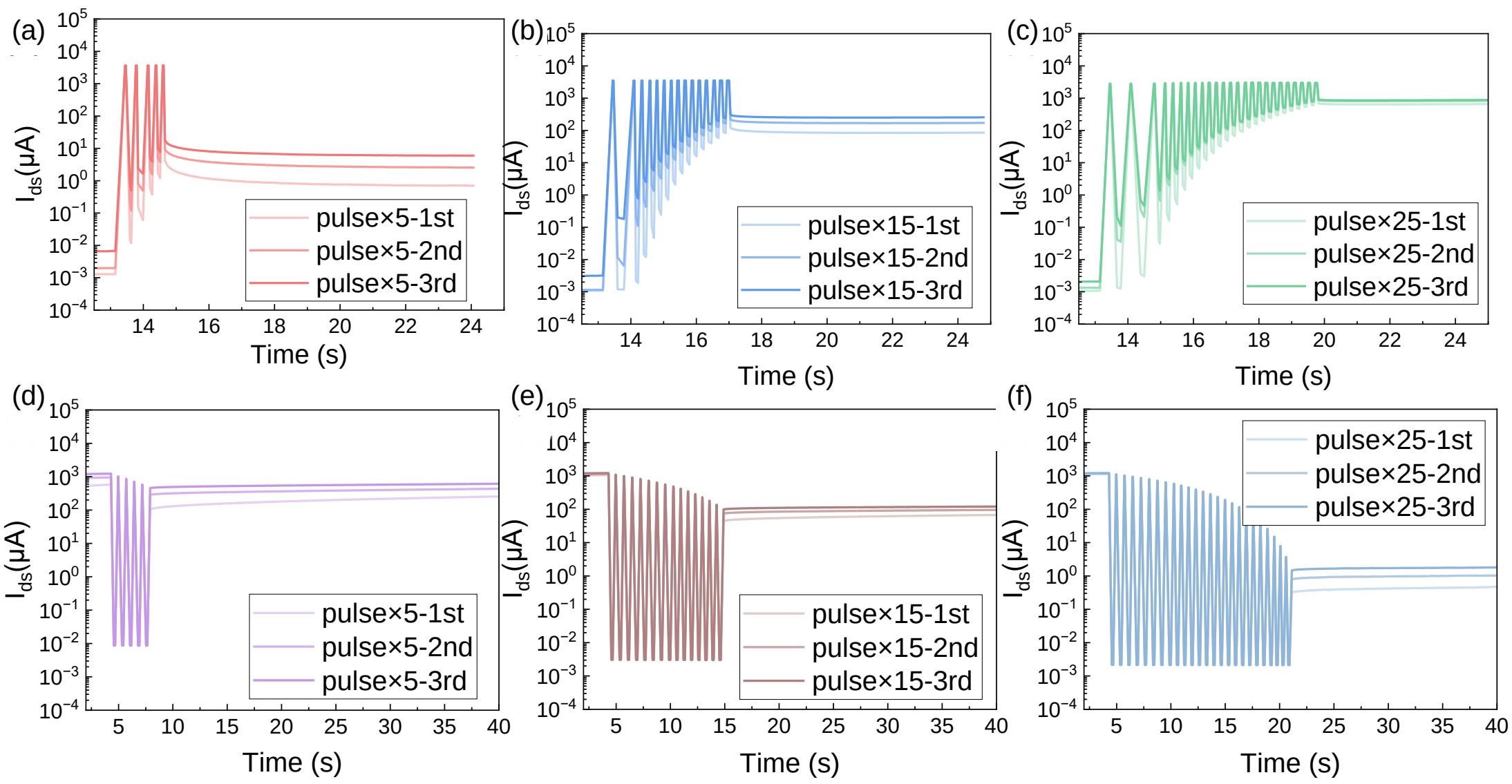

Supplementary Fig. 16. Long-Term Potentiation/Depression Characteristics of the *κ*-Ga_2O_3 FeHEMT. (a) Excitatory responses under 5 pulses. (b) Excitatory responses under 15 pulses. (c) Excitatory responses under 25 pulses. (d) Inhibitory responses under 5 pulses. (e) Inhibitory responses under 15 pulses. (f) Inhibitory responses under 25 pulses.

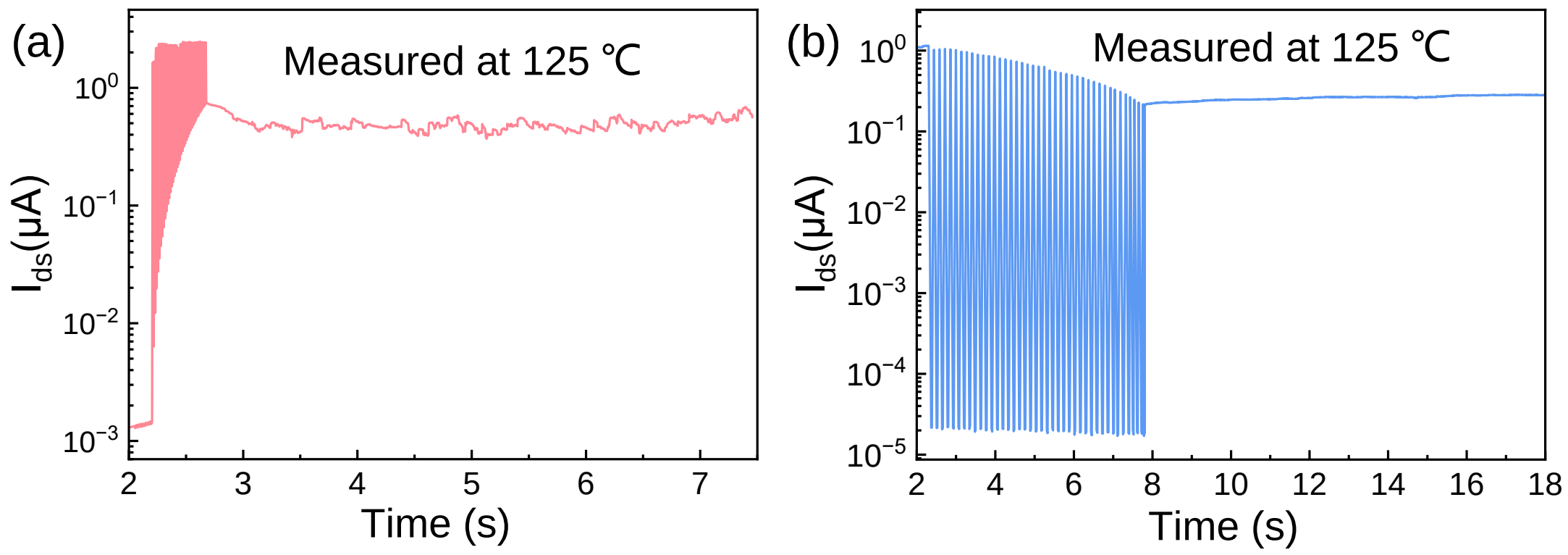

Supplementary Fig. 17. Long-term retention of κ-Ga_2O_3 FeHEMT at 125 ℃. (a) Long-term potentiation of κ-Ga_2O_3 FeHEMT at 125 ℃. (b) Long-term depression of κ-Ga_2O_3 FeHEMT at 125 ℃.

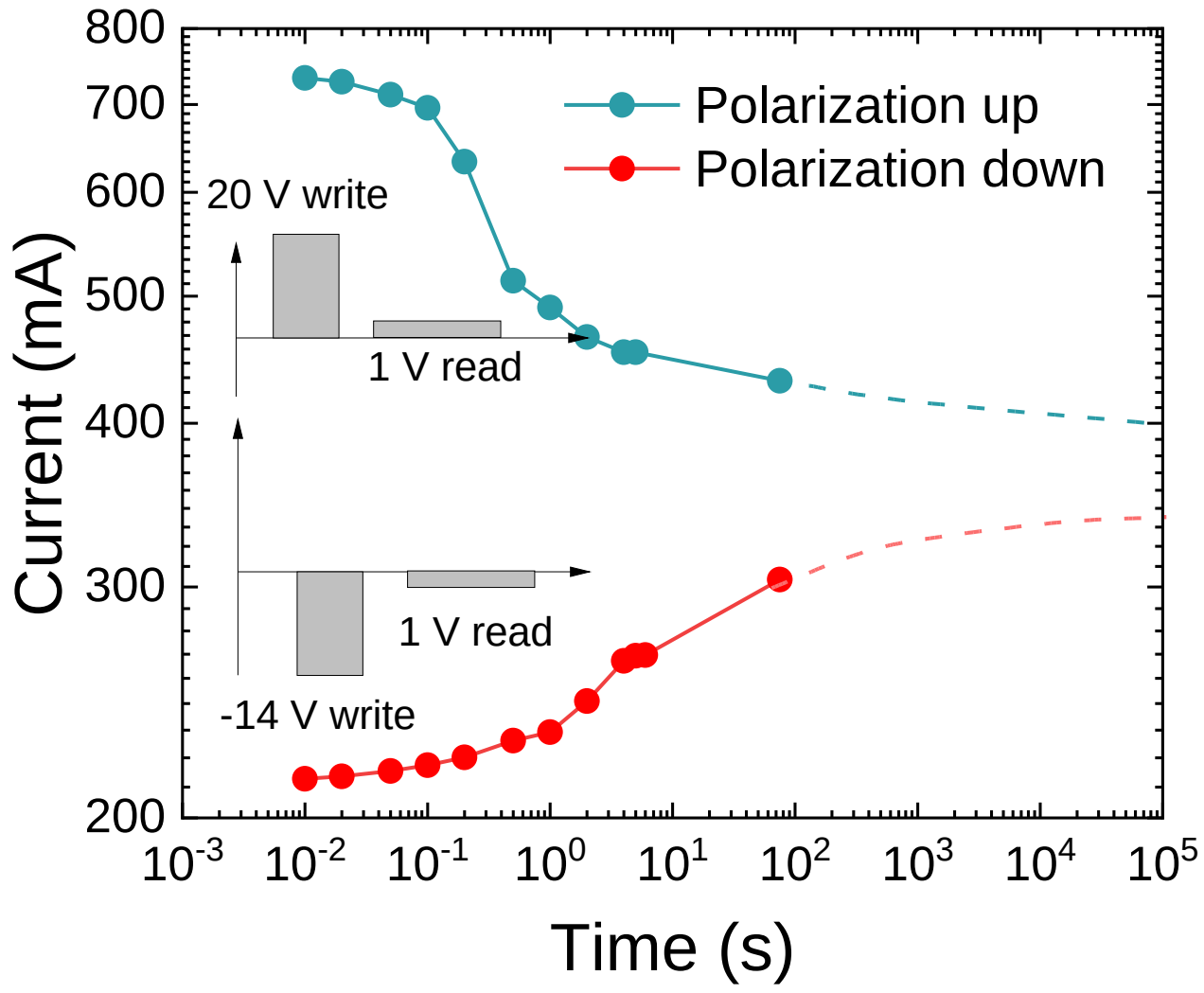

Supplementary Fig. 18. Retention measurement showing stable high and low resistance.

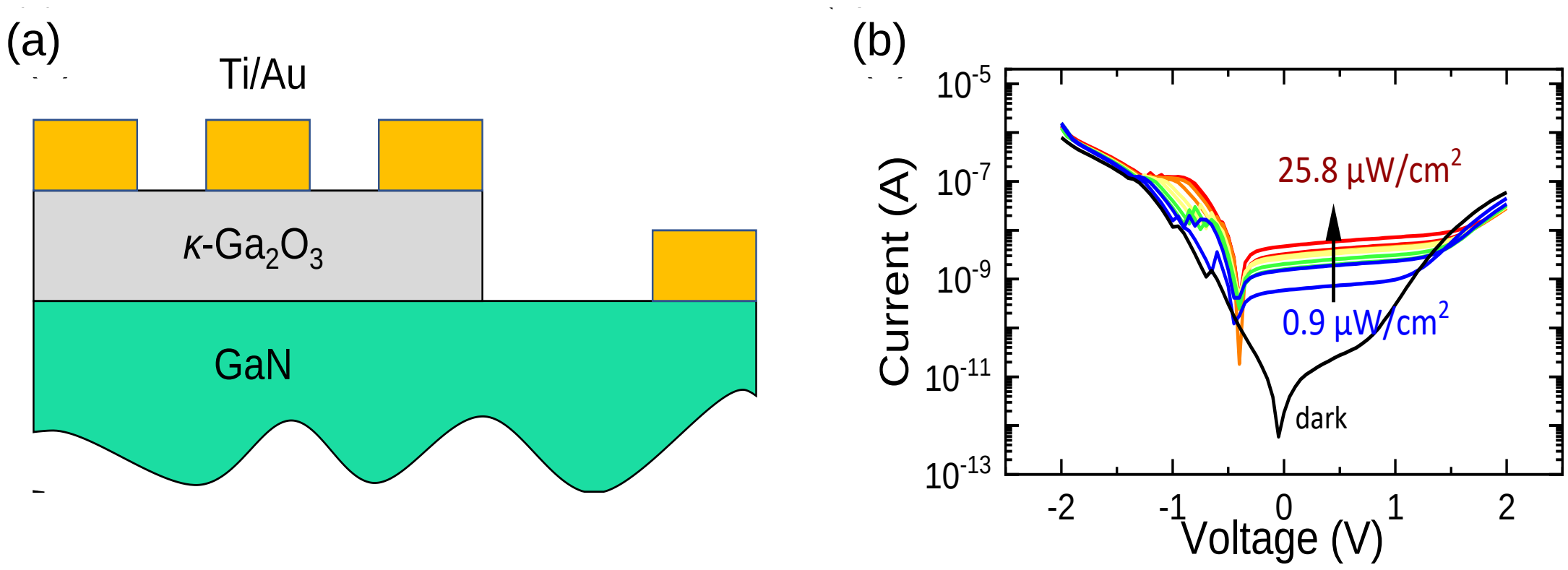

Supplementary Fig. 19. Optoelectronic characteristics of κ-Ga_2O_3-based DUV sensor. (a) Device structure of the DUV sensor. (b) *I-V* characteristics under varying incident intensities of the 261 nm light.

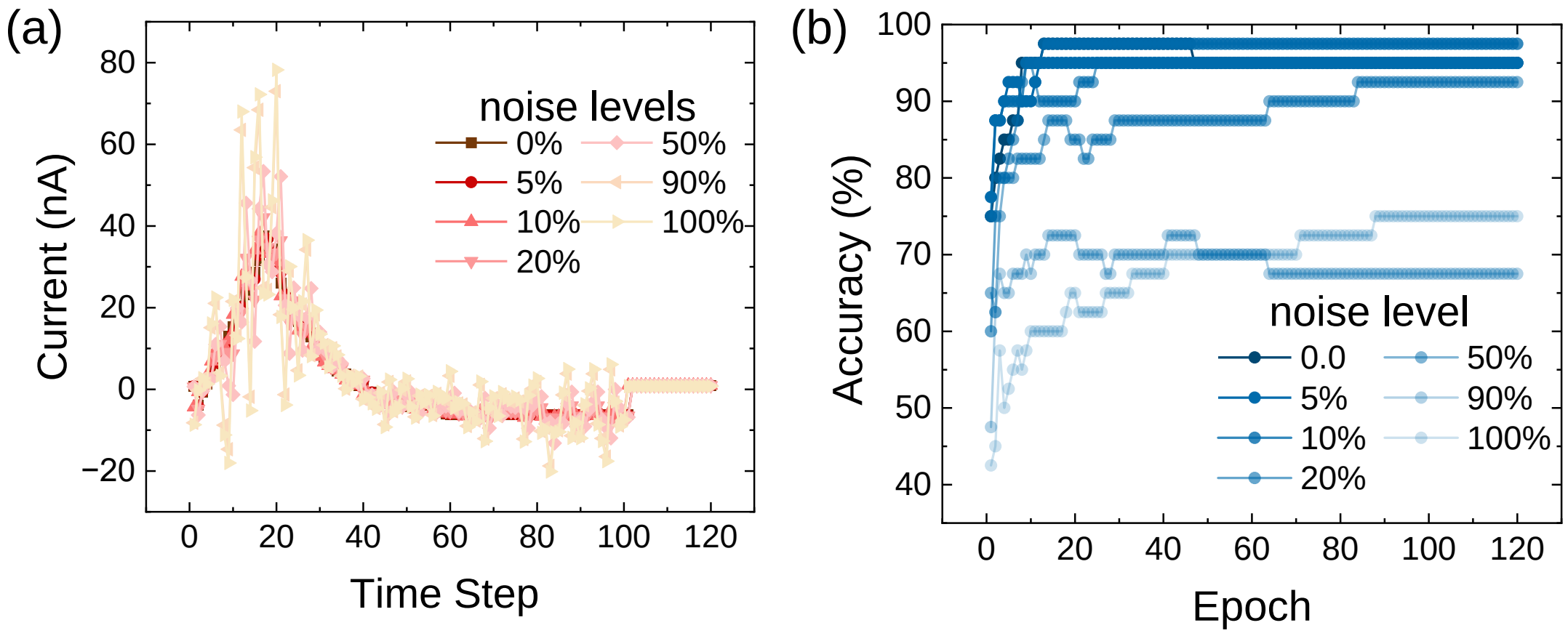

Supplementary Fig. 20. Noise-tolerance analysis of the FeHEMT-based reservoir computing system for celestial activity recognition. (a) Simulated current fluctuations under different noise levels. (b) Recognition accuracy as a function of training epoch under different noise levels.

Supplementary Table 1. Comparative summary among in-sensor reservoir computing devices with wide bandgap materials.

Device structure	System DUV hardness			λ (nm)	Intelligent tasks		Ref.
	Sensor	Reservoir	Synapse		Content	Radiation-resistance capability [a]	
ZnO/IGZO	×	×	×	365	Human action classification & MNIST image recognition	Medium	[7]
P(VDF-TrFE)/$Cs_2AgBiBr_6$	×	×	×	445	Face classification & dynamic vehicle flow recognition	Weak	[8]
$CeO_2/SrTiO_3$	×	×	×	375	Pattern recognition, Corona discharge localization	Weak	[9]
Pt/ZnO/Pt	×	×	×	365	Real-time pattern recognition	Medium	[10]
ITO/ZnO/MoO_3/SiO_2/Si	×	-	×	390	Image recognition	Medium	[11]
Pd/MoO_x/ITO	×	-	×	365	Visual memorizing, image recognition	Medium	[12]
GaN NWs sensor array	×	-	×	365	MNIST image recognition	Medium	[13]
GaO_x photo-synapses with TaO_x/HfO_y memristor array	√	√	√	254	Fingerprint recognition	High	[14]
Ga_2O_3/ZnO	-	-	×	265	MNIST image recognition	Medium	[15]
β-Ga_2O_3 transistor	√	-	√	-	Image recognition	High	[16]
κ-ISRC	√	√	√	261	Celestial activity	High	**This work**

[a] The radiation-resistance capability is intrinsically determined by the minimum bandgap in the **active-region** (E_g^{min}) participating in device operation under stress, with **High** for $E_g^{min} \geq 4$ eV, **Medium** for 3 eV$\leq E_g^{min}<4$ eV and **Weak** for $E_g^{min}<3$ eV. For example, AlGaN/GaN HEMTs sustain prolonged deep-UV (254–265 nm) illumination without bulk damage [17].

Supplementary Table 2. Comparison of device-level reservoir-state generation energy with representative embedded computing systems.

**Task**	**System**	**Energy consumption**	**Reference**
Celestial activities recognition	κ-ISRC	~15.6 mJ per action*	This work
Plant disease classification	Jetson Nano edge platform	~61.48 mJ per inference	[18]
Object detection / computer vision inference	Jetson Nano	~554 mJ per frame	[19]
Touch-modality classification	Custom hardware SVM	~81 mJ per inference	[20]
Image classification	Jetson Nano GPU	~530.72 mJ per inference	[21]
Speech / emergency-signal enhancement	STM32H735 MCU	~48.07 mJ per inference	[22]
HOG3D-based recognition system	FPGA implementation	~1940 mJ per action	[23]

* per action contains 100 reservoir states

References

1. Jónsson, H., Mills, G. & Jacobsen, K. W. Nudged elastic band method for finding minimum energy paths of transitions. In Classical and Quantum Dynamics in Condensed Phase Simulations (eds Berne, B. J., Ciccotti, G. & Coker, D. F.) World Scientific, Singapore, 1998).

2. Xiao, D., Chang, M.-C. & Niu, Q. Berry phase effects on electronic properties. *Rev. Mod. Phys*. **82**, 1959-2007 (2010).

3. Wong H S, White M H, Krutsick T J, Booth R V. Modeling of transconductance degradation and extraction of threshold voltage in thin oxide MOSFETs. *Solid-State Electron*. **30**, 953–968 (1987).

4. Aminbeidokhti A, Dimitrijev S, Hanumanthappa A K, Moghadam H A, Haasmann D, Han J, Shen Y, Xu X. Gate-voltage independence of electron mobility in power AlGaN/GaN HEMTs. *IEEE Trans. Electron Devices* **63**, 1013–1019 (2016).

5. Menozzi R, Umana-Membreno G A, Nener B D, Parish G, Sozzi G, Faraone L, Mishra U K. Temperature-dependent characterization of AlGaN/GaN HEMTs: Thermal and source/drain resistances. *IEEE Trans. Device Mater. Reliab.* **8**, 255–264 (2008).

6. Christopoulos, C. The transmission-line modeling method: TLM. *IEEE Antennas Propag.* Mag. **39**, 90–92 (1997).

7. Sun Y, et al. In-sensor reservoir computing based on optoelectronic synapse. *Adv. Intell. Syst.* **5**, 2200196 (2023).

8. Lao J, et al. Ultralow-power machine vision with self-powered sensor reservoir. *Adv. Sci.* **9**, 2106092 (2022).

9. Li G, et al. Interface-engineered non-volatile visible-blind photodetector for in-sensor computing. *Nat. Commun.* **16**, 57 (2025).

10. Jiang J, et al. Hardware-Level Image Recognition System Based on ZnO Photo-Synapse Array with the Self-Denoising Function. *Adv. Funct. Mater.* **34**, 2313507 (2024).

11. Guo T, et al. Broadband optoelectronic synapse enables compact monolithic neuromorphic machine vision for information processing. *Adv. Funct. Mater.* **33**, 2303879 (2023).

12. Zhou F, et al. Optoelectronic resistive random access memory for neuromorphic vision sensors. *Nat. Nanotechnol*. **14**, 776-782 (2019).

13. Du H, et al. GaN Nanowire nin Diode Enabled High-Performance UV Machine Vision System. *IEEE Trans. Nanotechnol*. **23**, 529–534 (2024).

14. Zhang Z, et al. In-sensor reservoir computing system for latent fingerprint recognition with deep ultraviolet photo-synapses and memristor array. *Nat. Commun*. **13**, 6590 (2022).

15. Wang J, et al. Piezo-phototronic effect modulated optoelectronic artificial synapse based on a-Ga_2O_3/ZnO heterojunction. *Nano Energy* **120**, 109128 (2024).

16. Wang X, et al. Ultra-low Power In-sensor Computing β-Ga_2O_3 Ultraviolet Optoelectronic Synaptic Devices. *IEEE Photonics Technol. Lett*. **36**, 1393-1396 (2024).

17. Wohlfahrt, M., Uren, M. J., Kaess, F., Laboutin, O., Hirshy, H. & Kuball, M. UV-induced change in channel conductivity in AlGaN/GaN high electron mobility transistors to measure doping. *Appl. Phys. Lett*. **118**, 163501 (2021).

18. Thaduri, A., Jog, M. C., Velmurugan, R. & Shojaei Baghini, M. Novel Grad-CAM based mask-guided attention for plant disease classification on edge. In *2024 IEEE International Conference on Omni-layer Intelligent Systems (COINS)*, 1-6 (IEEE, 2024).

19. Machado, P., Matic, I., de Lemos, F., Ihianle, I. K. & Adama, D. A. Estimating the power

consumption of heterogeneous devices when performing AI inference. arXiv 2207.06150 (2022).
20. Alameh, M., Abbass, Y., Ibrahim, A. & Valle, M. Smart tactile sensing systems based on embedded CNN implementations. *Micromachines* **11**, 103 (2020).
21. Ning, Z., Vandersteegen, M., Van Beeck, K., Goedemé, T. & Vandewalle, P. Power consumption benchmark for embedded AI inference. In *Proceedings of the International Conferences on Applied Computing 2024 and WWW/Internet 2024* (2024).
22. Giorgetti, G. & Pau, D. P. Transitioning from TinyML to edge GenAI: a review. *Big Data Cogn. Comput.* **9**, 61 (2025).
23. Cui, H., Xiao, Y., Yang, Y., Pei, M., Ke, S., Fang, X., Qiao, L., Shi, K., Long, H., Xu, W., Cai, P., Lin, P., Shi, Y., Wan, Q. & Wan, C. A bioinspired in-materia analog photoelectronic reservoir computing for human action processing. *Nat. Commun.* **16**, 2263 (2025).
